# Factors linked to depressive symptoms in obsessive-compulsive disorder: a machine learning and network analysis from China OCD Cohort (COCC)

**DOI:** 10.1186/s12888-026-07854-z

**Published:** 2026-01-31

**Authors:** Yu Wu, Jieling Xu, Huan Zhang, Ping Zhou, Chenchen Shao, Xiaolu Zhang, Wenxin Tang, Qianqian Li, Jun Yan, Si Mi, Zhanjiang Li, Bin Li, Guiyun Xu, Congwen Yang, Maorong Hu, Zhenqing Zhang, Yanbin Jia, Zhen Tang, XiaoPing Wang, Jun Ma, Changhong Wang, Wei Liu, Na Liu

**Affiliations:** 1 Nanjing Brain Hospital Affiliated to Nanjing Medical University, Nanjing, Jiangsu China; 2 Department of Medical Psychology, The Affiliated Brain Hospital of Nanjing Medical University, Nanjing, Jiangsu China; 3 Affiliated Mental Health Center & Hangzhou Seventh People’s Hospital, Zhejiang University School of Medicine, Hangzhou, Zhejiang China; 4 Peking University Sixth Hospital, Peking University Institute of Mental Health, NHC Key Laboratory of Mental Health (Peking University), National Clinical Research Center for Mental Disorders (Peking University Sixth Hospital), Beijing, China; 5 Center of Clinical Psychology, Beijing Anding Hospital, Capital Medical University; National Clinical Research Center for Mental Disorders & National Center for Mental Disorders, Beijing Key Laboratory of Mental Disorders, Beijing, China; 6 Mental Health Center, West China Hospital, Sichuan University, Chengdu, Sichuan China; 7 The Brain Hospital Affiliated to Guangzhou Medical University, Guangzhou, Guangdong China; 8 School of Psychology, Guizhou Normal University, Guiyang, Guizhou China; 9 The 1st Affiliated Hospital, Jiangxi Medical College, Nanchang University, Nanchang, Jiangxi China; 10 Xiamen Xianyue Hospital, Xianyue Hospital Affiliated with Xiamen Medical College, Fujian Psychiatric Center, Fujian Clinical Research Center for Mental Disorders, Xiamen, Fujian China; 11 Department of Psychiatry, The First Affiliated Hospital of Jinan University, Guangzhou, Guangdong China; 12 The Affiliated Guangji Hospital of Soochow University, Suzhou, Jiangsu 215137 China; 13 Department of Psychiatry, National Clinical Research Center for Mental Disorders, The Second Xiangya Hospital of Central South University, Changsha, Hunan 410011 China; 14 Pediatric and Adolescent Health Ward, Wuhan Mental Health Center, Wuhan, Hubei China; 15 Henan Collaborative Innovation Center of Prevention and Treatment of Mental Disorder, The Second Affiliated Hospital of Henan Medical University, Zhengzhou, Henan China; 16 Department of Psychiatry, The First Affliated Hospital of Harbin Medical University, Harbin, Heilongjiang China

**Keywords:** Obsessive-compulsive disorder, Depression, Machine learning, Network analysis

## Abstract

**Background:**

Depressive symptoms are highly prevalent in individuals with obsessive-compulsive disorder (OCD) and substantially complicate clinical management. However, the feature structure associated with depressive symptoms in OCD remains insufficiently characterized, particularly from an integrative, multivariate perspective. This study aimed to identify key features associated with depressive symptoms in OCD and to elucidate their interrelationships using machine learning and network analysis.

**Methods:**

A multicenter sample of 1,293 patients with OCD was recruited from 15 specialized OCD clinics across China. An extreme gradient boosting (XGBoost) model was developed to predict depressive symptoms, with hyperparameter optimization conducted using Optuna and feature contributions quantified via SHAP values. Multivariable logistic regression was used to examine independent associations, and network analysis was applied to explore the co-occurrence structure among key features.

**Results:**

The XGBoost model identified anxiety, psychosocial functioning, mental state, obsessing, functional impairment, perceived stress, and gender as the most informative features associated with depressive symptoms in OCD. SHAP analyses indicated that higher anxiety levels, poorer psychosocial functioning, and a more negative self-rated mental state contributed most strongly to increased predicted risk. Network analysis further demonstrated that anxiety, mental state, obsessing, and psychosocial functioning occupied central positions within the network. Anxiety showed prominent bridging properties, exhibiting strong associations with obsessing, perceived stress, and mental state, suggesting its integrative role across emotional, cognitive, and functional domains.

**Conclusions:**

Depressive symptoms in OCD are embedded within a tightly interconnected configuration of emotional, cognitive, and functional features, with anxiety occupying a central and bridging position across analytical approaches. The combined application of machine learning and network analysis provides a complementary framework for identifying salient features and elucidating their interrelationships in OCD patients with depressive symptoms, with potential implications for clinical assessment and targeted intervention.

**Supplementary Information:**

The online version contains supplementary material available at 10.1186/s12888-026-07854-z.

## Introduction

Obsessive-Compulsive Disorder (OCD) is a common chronic mental health condition, characterized by persistent, intrusive obsessions (unwanted, repetitive thoughts) and/or compulsive behaviors (ritualistic actions performed to alleviate distress). The 12-month prevalence of OCD is estimated to range from 0.7% to 1.2% [1], with lifetime prevalence of 1% − 3% [2–4]. In China, the lifetime prevalence of OCD has been reported to be 2%-2.9% [5]. OCD imposes substantial disease burden with high disability rates, and has been classified as one of the top ten disabling medical conditions by the World Health Organization [6–8].

Comorbid psychopathology in OCD has been consistently associated with poorer clinical outcomes [4, 9, 10]. Among these conditions, major depressive disorder shows the highest co-occurrence with OCD [11], with depressive symptoms exhibiting heightened prevalence among OCD populations [12, 13]. Epidemiological studies consistently demonstrate lifetime prevalence of depression in OCD patients ranges from 40% to 80% [12, 14–16]. Empirical evidence confirms that depressive symptoms not only co-occur with greater severity of core OCD symptoms but are also associated with a longer illness course [17–19], while markedly reduced quality of life [20]. Individuals with OCD who exhibit elevated depressive symptoms also tend to present with higher levels of anxiety, poorer social functioning, and occupational maladjustment [17, 21]. Notably, depressive symptoms may compromise the therapeutic efficacy of core interventions such as Exposure and Response Prevention (ERP) therapy [22–24], and are associated with poorer treatment response and prognosis overall [25, 26]. In our previous neuroimaging work, we observed that greater depressive symptom severity in OCD was associated with reduced nodal efficiency and degree centrality of the right superior temporal gyrus (STG), which were in turn correlated with impairments in subjective well-being and cognitive functioning [27]. Although these findings do not imply causal relationships, they suggest that depressive symptoms in OCD are embedded within broader clinical and neurobiological profiles rather than representing an isolated comorbid condition. Taken together, these observations highlight the clinical importance of characterizing depression-related symptom patterns and associated factors within OCD, and underscore the need for integrative analytical approaches to identify individuals at elevated risk for clinically significant depressive symptoms.

Despite extensive investigations into the relationship between OCD and depressive symptoms, significant research gaps remain. Most prior studies have relied on descriptive approaches that evaluate individual correlates of depression in isolation, offering limited insight into the relative importance of multiple, interrelated clinical and psychosocial factors within OCD populations [15]. Although network analysis has been employed to examine symptom hierarchies, such investigations are constrained by restricted sample characteristics (specific age groups/populations) and psychometric limitations in assessment tools [16]. Current assessment paradigms predominantly operate at the scale-item level, lacking integration of symptom hierarchy analyses, which restricts multidimensional investigation [14]. Crucially, previous research has adopted fragmented perspectives, focusing either on OCD severity or symptom typology, without establishing a multidimensional integrative analytical framework [18].

Recent advances in machine learning have provided powerful tools for identifying clinically relevant features from high-dimensional psychiatric data, particularly in contexts where multiple interrelated variables jointly contribute to symptom burden [19, 28]. The Extreme Gradient Boosting (XGBoost) algorithm, being recognized for its computational efficiency and flexibility, has demonstrated robust capacity in processing large-scale multidimensional clinical datasets [29, 30]. Previous research has also confirmed the performance of the XGBoost model in clinical studies [31–33]. To enhance the interpretability of the predictive model, SHapley Additive exPlanations (SHAP) were employed alongside XGBoost. SHAP provides a unified framework for quantifying the contribution of each feature to individual model predictions, thereby enabling the assessment of feature importance and directionality. In this context, machine learning–based modeling primarily serves to prioritize depression-related features according to their predictive relevance within a multivariate setting. Furthermore, network analysis has emerged as a novel psychometric approach that conceptualizes symptom associations as network structures, with symptoms serving as nodes and their relationships as the connecting edges. This data-driven methodology characterizes symptoms as interconnected nodes, eschewing predefined causal assumptions [34, 35], and offers an alternative framework for examining the configurational organization and statistical co-occurrence patterns of depressive symptom–related features through quantitative evaluation of association structures [20]. By situating machine learning–derived feature prioritization within a network-based analytical framework, this integrative approach allows predictive relevance to be complemented by descriptive structural characterization, offering a coherent strategy for examining complex, multivariate depression-related feature profiles.

The present study implemented a national multi-center design to collect multi-dimensional clinical datasets of China OCD Cohort. Using Extreme Gradient Boosting (XGBoost) with SHapley Additive exPlanations (SHAP), we identified and ranked variables most strongly associated with elevated depressive symptoms in individuals with OCD. Network analysis was subsequently applied to characterize the relational organization among these depression-related factors. By integrating predictive modeling with network-based analysis, this study provides a systematic, multivariate characterization of depression-related profiles in OCD, with potential implications for risk stratification and the generation of testable hypotheses for future research.

## Methods

### Participants

This study enrolled multicenter participants with obsessive-compulsive disorder (OCD) across 15 psychiatric hospitals or psychiatric departments of general hospitals, covering major regions in northern, central, eastern, and southern China and established China Obsessive-Compulsive Disorder Cohort-Chinese Obsessive-Compulsive Collaborative (China OCD Cohort-COCC), mainly clinical treatment-seeking outpatients and inpatients. The specific inclusion criteria included (1) meeting the Diagnostic and Statistical Manual of Mental Disorders (DSM-5) criteria for OCD, (2) age above 17 years, (3) minimum junior high school education with literacy and capacity to complete questionnaires and assessments. Exclusion criteria included: (1) having a mental disorder due to medical conditions or substance use, (2) history of intellectual disability, encephalitis, epilepsy, traumatic brain injury, neurological disorders, or neurosurgery, (3) Comorbid schizophrenia spectrum disorders, bipolar disorder, or other mental disorders that meet the DSM-5 diagnostic criteria, (4) severe physical comorbidities. All participants were diagnosed by senior psychiatrists and assessed after providing informed consent. The final cohort comprised 1,293 patients. This study received ethical approval from the affiliated Nanjing Brain Hospital of Nanjing Medical University (2021-KY044-01). All participants signed informed consent documents.

### Sociodemographic questionnaire

The investigator-developed assessment instrument collected comprehensive data across multiple domains, including social demographic characteristics (age, gender, occupation, and marital status), early life experiences, social support and current living conditions, among others.

### Clinical features, psychological assessments, and functional assessments

#### Yale-Brown Obsessive-Compulsive Scale (Y-BOCS) and obsessive compulsive inventory-revised (OCI-R)

To assess obsessive-compulsive symptoms, this study utilized the Yale-Brown Obsessive-Compulsive Scale (Y-BOCS) [36], recognized as the gold standard for evaluating the severity and symptoms of OCD. This 10-item instrument quantifies temporal expenditure, functional interference, subjective distress, resistance attempts, and perceived control regarding obsessions (items 1–5) and compulsions (items 6–10) during the preceding 30-day period. The scale follows a 5-point scoring system, ranging from 0 to 40, has demonstrated satisfactory psychometric properties, with elevated scores indicating greater symptom severity. The Chinese version of Y-BOCS showed good reliability with a consensus reliability of 0.75 [37].

Developed by Professor Foa et al. [38] based on the simplification of the original OCI, the OCI-R consists of 18 items that assess patients’ obsessive-compulsive symptoms over the past month across 6 dimensions: Washing, Obsessing, Hoarding, Ordering, Checking, and Neutralizing. Each dimension contains 3 items. The scale is scored on a 0–4 basis, with a total score range of 0–72. The Chinese version demonstrates good internal consistency, with a Cronbach’s α coefficient of 0.88 [39].

#### Beck Depression Inventory-II (BDI-II)

The Chinese version of the Beck Depression Inventory-II (BDI-II) [40] was employed to evaluate the severity of depressive symptoms. This 21-item instrument utilizes a 0–3 Likert scale, with total scores ranging from 0 to 63 (0–13: minimal depression; 14–19: mild; 20–28: moderate; 29–63: severe) [41]. Participants with scores of 14 or higher were assigned to the depressive subgroup. The scale exhibited excellent internal consistency (Cronbach’s α = 0.94). The Mandarin Chinese adaptation of the depression scale has shown an internal consistency as measured by a Cronbach’s α coefficient of 0.94 [42].

#### Beck Anxiety Inventory (BAI)

The Chinese version of the Beck Anxiety Inventory (BAI) assesses anxiety severity using a 21-item scale with each item scored from 0 to 3. The total score ranges from 0 to 63, with established severity thresholds: 0–7 (minimal), 8–15 (mild), 16–25 (moderate), and ≥ 26 (severe). This instrument demonstrated excellent internal consistency (Cronbach’s α coefficient = 0.95) [43].

#### Psychosocial functioning

The Sheehan Disability Scale (SDS) [44] measured functional impairment across occupational/educational, social/leisure, and family domain (0–10 per item; 0: no impairment; 1–3: mild; 4–6: moderate; 7–10: severe impairment). The total scores ranged from 0 to 30, with higher scores indicating more significant disability. Psychosocial Functioning Questionnaire [45] assessed three dimensions (subjective well-being, psychological cognition, social functioning) using a 5-point Likert scale (total range: 0–90; higher scores=greater impairment). It demonstrated robust psychometric properties (Cronbach’s α = 0.842–0.945).

#### Perceived Stress Scale (PSS)

The Perceived Stress Scale (PSS) [46] consists of 10 items, each rated on a 0 to 4 scale. The total score ranges from 0 to 40, with higher scores indicating a higher level of stress perceived by the individual over the past month. The Cronbach’s α coefficient of the Chinese version scale was 0.91, indicating good internal consistency [47]. 

### Overview of the analytical strategy

A multi-stage analytical framework was applied in this study. First, machine learning techniques were used to identify key factors associated with depressive symptoms from high-dimensional features. Second, multivariable logistic regression analyses were conducted to examine the independent associations between these factors and depressive status. Finally, network models were constructed to explore the interrelationships among the identified factors.

### Data preprocessing and machine learning procedures

Clinically significant depressive symptoms were defined using a cutoff score of BDI-II ≥ 14 [41]. The dataset was randomly divided into a training set and a testing set in a 7:3 ratio, with stratified sampling applied to preserve the proportion of participants with depressive symptoms across the two subsets. A small proportion of missing data (< 2%) was handled using single imputation, with the mode applied for categorical variables and the mean for continuous variables. To address mild class imbalance (55.7% of participants classified as having depressive symptoms), the SMOTE technique was applied to the training dataset [48]. Feature selection was performed using recursive feature elimination with cross-validation (RFECV) [49], and hyperparameter optimization was conducted using the Optuna framework [50] (see Supplementary Methods S1. for details). Model performance was evaluated in the testing dataset, and model interpretability was assessed using SHAP values [51]. To examine robustness, results from the classification model (binary depressive status) were compared with those obtained from a regression model using continuous BDI-II total scores (see Supplementary Figure S5. for details).

### Statistical validation and network analysis

Key factors identified through machine learning were entered into multivariable logistic regression models to estimate their associations with depressive status, expressed as odds ratios (ORs). An association network was subsequently constructed based on these factors using graphical LASSO regularization to examine their interrelationships. Network stability was evaluated using bootstrap procedures [52] (see Supplementary Methods S2. for details).

### Statistical and programming software

**Fig. 1:**
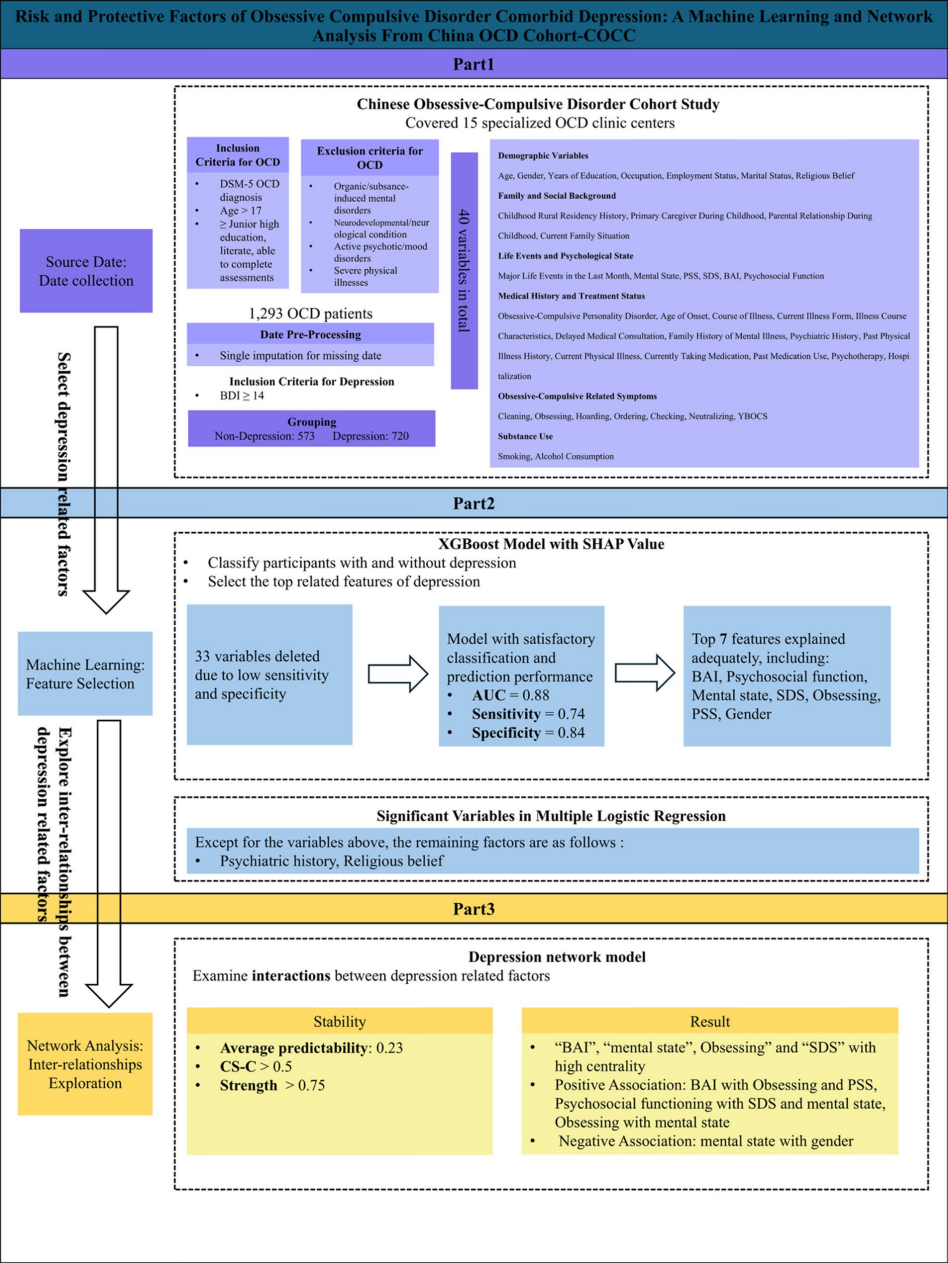
Machine learning and network analysis integration flowchart

The analyses were conducted utilizing Python 3.11.9 (Python Software Foundation, PSF) and R 4.4.1 (R Foundation for Statistical Computing, RFC). Statistical analysis was performed using SPSS 27.0. Continuous variables are presented as mean ± standard deviation (SD), while categorical variables are presented as frequencies with percentages (n, %). A binary logistic regression analysis was conducted, with depression in OCD patients as the dependent variable, incorporating all study variables to evaluate the statistical significance of both model-specific and extraneous factors. To clarify the implementation of our study, Fig. 1. presents the entire experimental process.

## Results

### Demographic and clinical characteristics

A total of 1,293 patients with obsessive–compulsive disorder were included (Table 1.). Of these patients, 55.7% met criteria for clinically significant depressive symptoms (BDI-II ≥ 14). The mean age was 28.72 years (SD = 9.19), 56.5% were male, the mean age at OCD onset was 21.74 years (SD = 8.91), and the mean illness duration was 6.97 years (SD = 6.45). A detailed description of all clinical and demographic characteristics is provided in Supplementary Table S1.

### XGBoost derivative model and performance

**Fig. 2:**
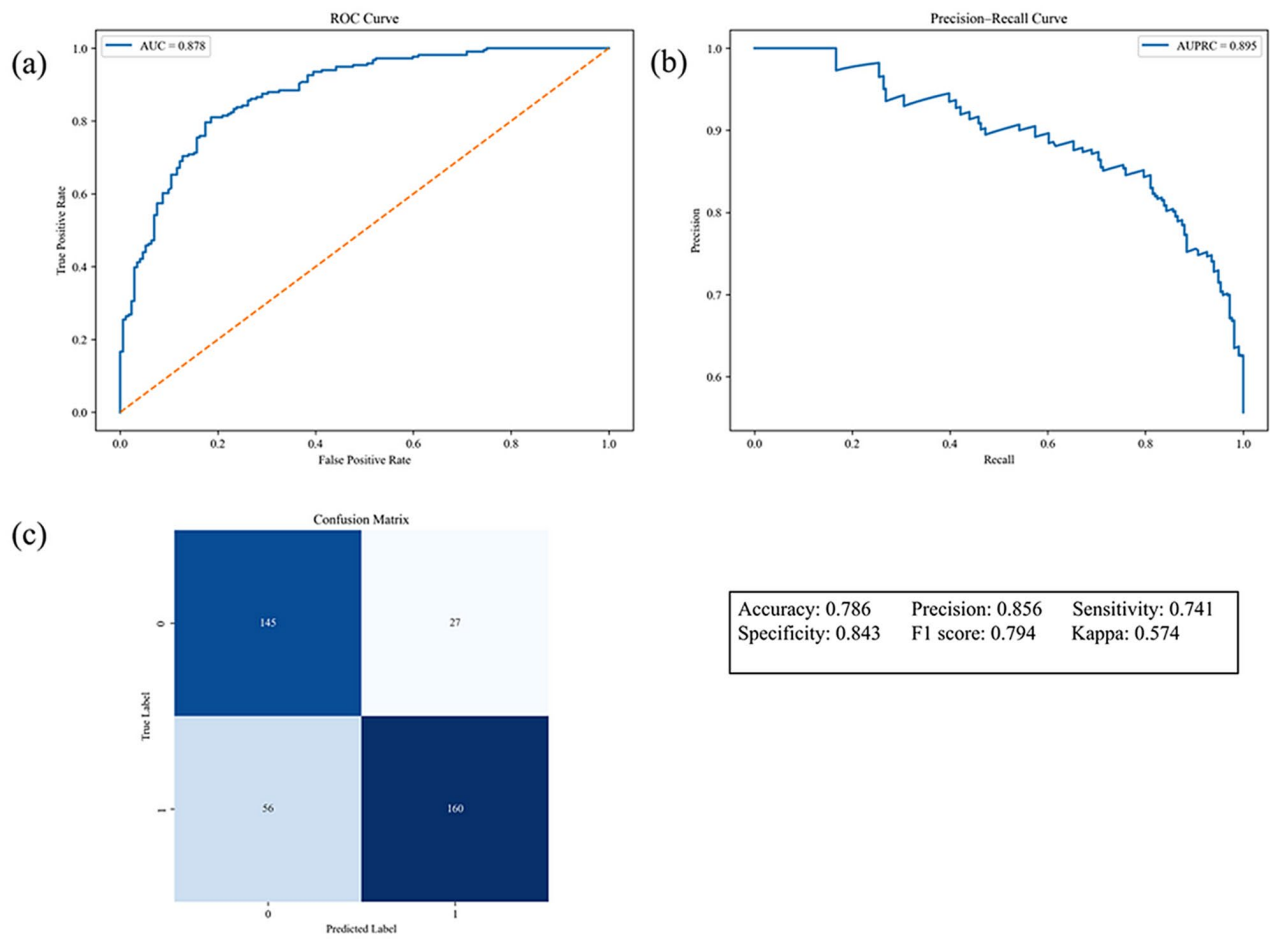
Performance evaluation of the XGBoost model. **(a)** Weighted receiver operating characteristic (ROC) curve illustrating the trade-off between sensitivity and specificity across different probability thresholds. **(b)** Precision–recall (PR) curve assessing model performance under class imbalance conditions. **(c)** Confusion matrix of the XGBoost model evaluating classification accuracy for patients with and without depressive symptoms; accuracy, recall, and F1 score were derived from the matrix to further characterize predictive performance

**Fig. 3:**
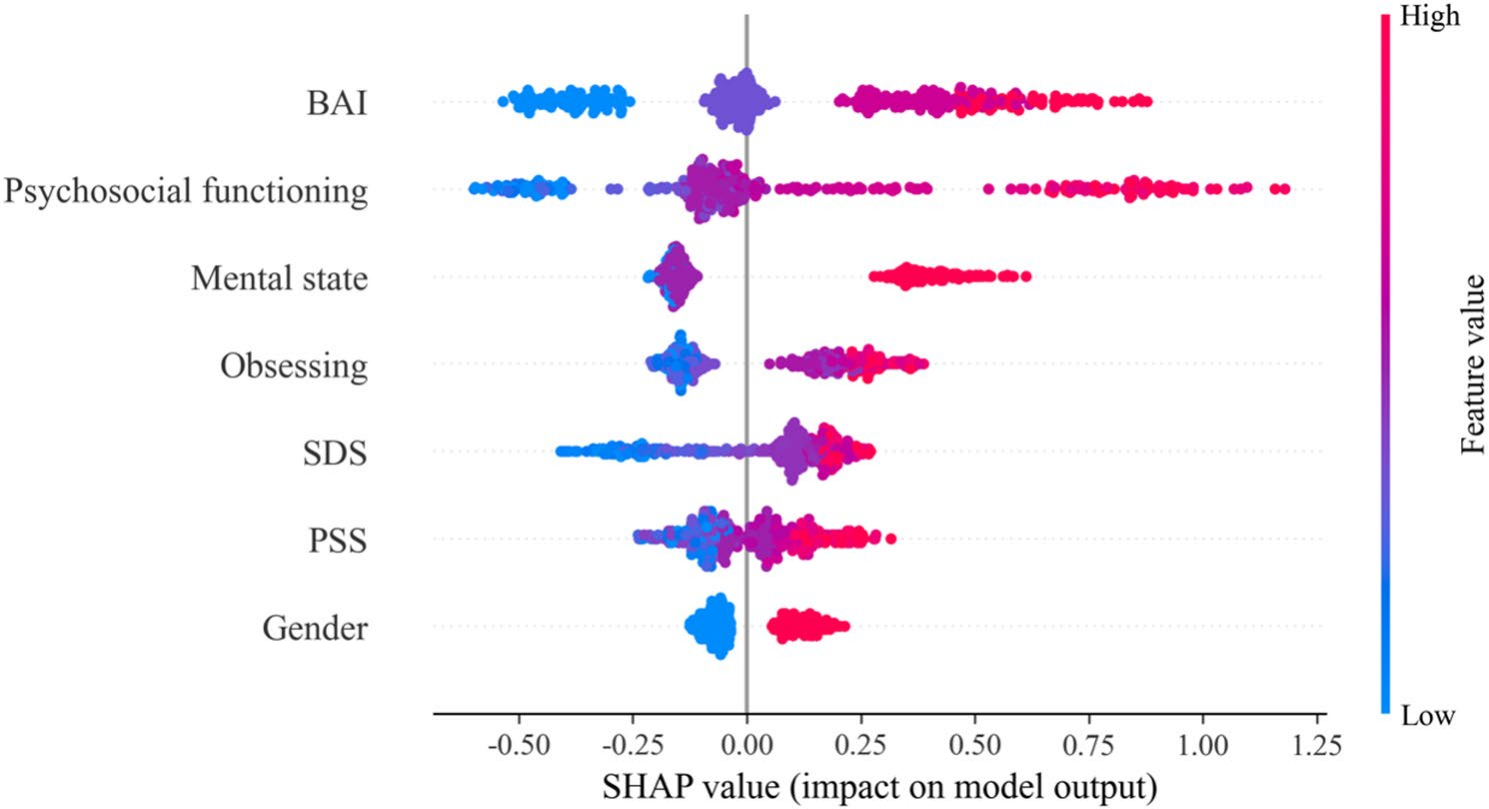
SHAP-based summary plot of key predictive features. Features are ranked in descending order according to their mean absolute SHAP values. Point color indicates the original feature value (blue = low, red = high), and the horizontal position reflects the direction of the feature’s impact on model predictions (right = positive, left = negative). Notes: BAI: Beck Anxiety Inventory; SDS: Sheehan Disability Scale; PSS: Perceived Stress Scale

To identify key predictors of depressive symptoms, an XGBoost model with optimized hyperparameters was developed and evaluated in an independent test set. The model demonstrated good discriminative performance, with an area under the receiver operating characteristic curve (AUC) of 0.878 (Fig. 2a.) and an area under the precision–recall curve (AUPRC) of 0.8959 (Fig. 2b.). At the optimal decision threshold (Fig. 2c.), the model achieved an accuracy of 0.786, sensitivity of 0.741, and specificity of 0.843. Model performance was stable across repeated random data splits, with a mean AUC of 0.879 ± 0.014 over 10 iterations (Supplementary Fig S1.). SHAP-based interpretation identified the most influential features associated with depressive symptoms, including BAI, psychosocial functioning, mental state, obsessing, SDS, PSS, and gender (Fig. 3.) (Detailed SHAP dependence plots are presented in Supplementary Fig S2–S4). Sensitivity analyses using a regression model with continuous BDI-II scores showed highly consistent feature importance rankings with the classification model (Spearman’s ρ = 0.929; Supplementary Fig S5), supporting the robustness of the findings.

### Statistical validation

Multivariable logistic regression confirmed the independent associations of the identified key factors with depressive symptoms (Table 1). Anxiety symptoms showed the strongest association (OR = 9.37, 95% CI: 4.93–17.79). Other variables that remained significant included psychosocial functioning, self-reported mental state, functional impairment, obsessing, and male sex (full regression results are provided in Supplementary Table S1).

**Table 1:** Comparison of the related factors of depressive symptoms in patients with obsessive-compulsive disorder and logistic regression analysis

Independent variable	Non- Depression (*n* = 573)	Depression (*n* = 720)	Odds ratio (95% CI)	β
Gender (Ref: female)				
Male	356(62.1%)	375(52.1%)	0.43(0.30∽0.62)	-0.837^***^
Religious Belief (Ref: None)				
Yes	27(4.7%)	45(6.3%)	0.44(0.21∽0.90)	-0.831^*^
Mental State (Ref: Good)				
Poor	84(14.7%)	374(51.9%)	3.56(1.86∽6.82)	1.27^***^
Neutral	366(63.9%)	320(44.4%)	1.55(0.86∽2.81)	0.44
PSS	19.43 ± 4.36	22.42 ± 3.89	1.05(1.00∽1.10)	0.046^*^
Psychiatric History (Ref: None)				
Yes	216(37.7%)	333(53.2%)	0.65(0.47∽0.89)	-0.435^**^
OCIR				
Cleaning	3.74 ± 3.51	5.12 ± 3.94	1.00(0.95∽1.05)	-0.004
Obsessing	4.28 ± 2.79	7.00 ± 2.89	1.08(1.01∽1.16))	0.078^*^
Hoarding	1.81 ± 2.17	2.81 ± 2.58	1.04(0.96∽1.12)	0.035
Ordering	2.54 ± 2.47	3.95 ± 3.03	1.05(0.97∽1.13)	0.046
Checking	3.71 ± 3.05	5.07 ± 3.28	0.98(0.93∽1.04)	-0.02
Neutralizing	2.38 ± 2.62	3.70 ± 3.12	1.00(0.94∽1.07)	0
Psychosocial Function	49.75 ± 8.92	59.18 ± 8.24	1.11(1.08∽1.14)	0.104^***^
SDS	12.12 ± 6.58	18.21 ± 6.01	1.07(1.04∽1.11)	0.07^***^
BAI(Ref: None)				
Severe	21(3.7%)	192(26.7%)	9.37(4.93∽17.79)	2.237^***^
Moderate	87(15.2%)	244(33.9%)	4.33(2.74∽6.84)	1.466^***^
Mild	174(30.4%)	210(29.2%)	2.73(1.82∽4.11)	1.004^***^

Notes: PSS: Perceived Stress Scale; YBOCS: Yale-Brown Obsessive-Compulsive Scale; SDS: Sheehan Disability Scale; BAI: Beck Anxiety Inventory. The numbers outside and inside the parentheses represent the sample size and percentage (%) for the “non-depressed group” and the “depressed group,” respectively. The β represents the regression coefficient. *∽p < 0.05.**∽p < 0.01.***∽p < 0.001

### Results of the network model

**Fig. 4:**
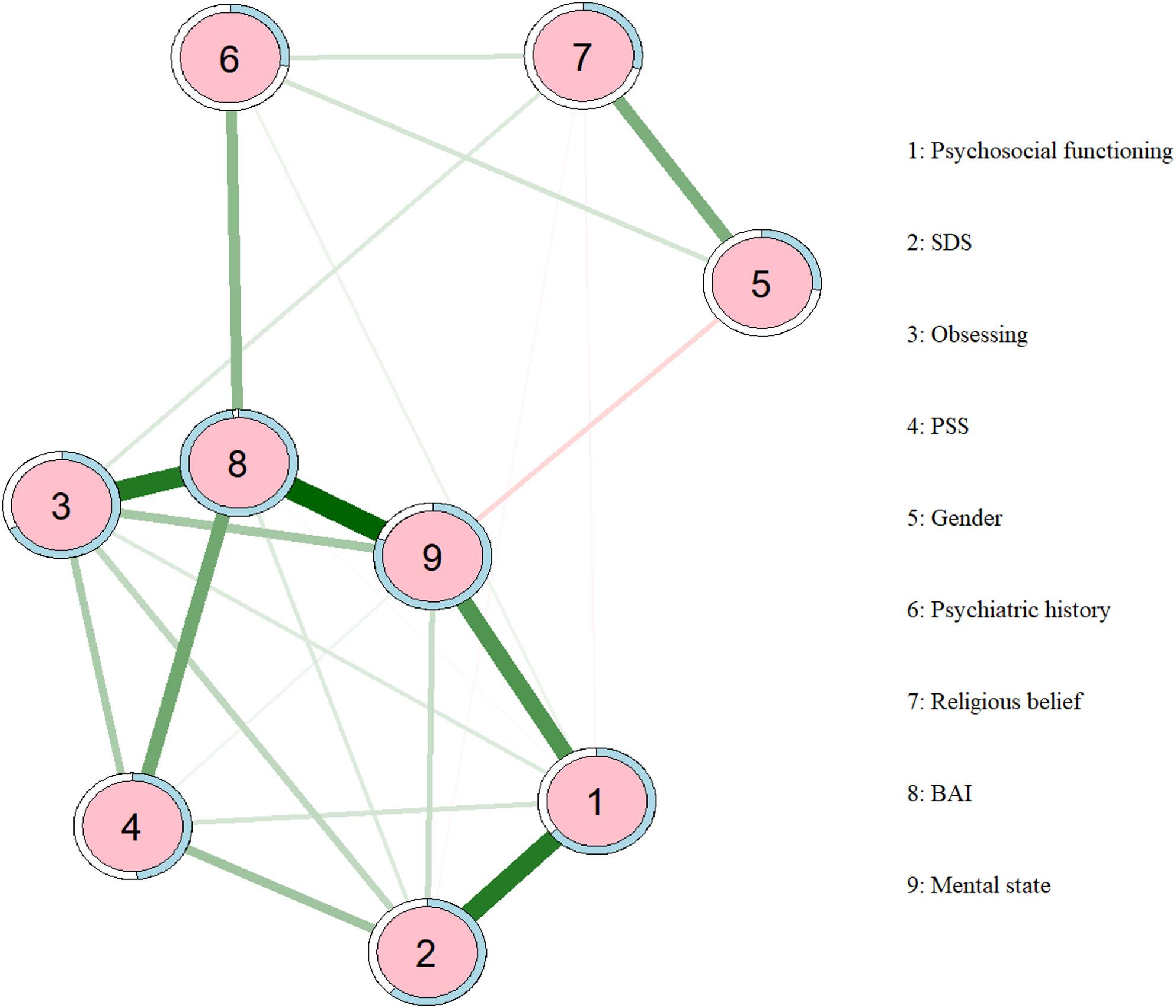
Network analysis of depression-related factors. Green edges represent positive correlations, while red edges indicate negative correlations. Notes: BAI: Beck Anxiety Inventory; SDS: Sheehan Disability Scale༛PSS: Perceived Stress Scale

Variables identified as significant in the XGBoost model and multivariable regression were entered into the network analysis. As shown in Fig. 4., the estimated network of depression-related features comprised nine nodes, including psychosocial functioning, SDS, obsessing, PSS, gender, psychiatric history, religious belief, BAI, and self-reported mental state. Within the network, the strongest positive associations (indicated by the darkest green edges) were observed between obsessing and anxiety, anxiety and mental state, mental state and psychosocial functioning, and mental state and SDS. In contrast, mental state showed a negative association with gender. Overall, anxiety and mental state emerged as central nodes, displaying strong connections with multiple variables and occupying key positions within the psychological feature network.

**Fig. 5:**
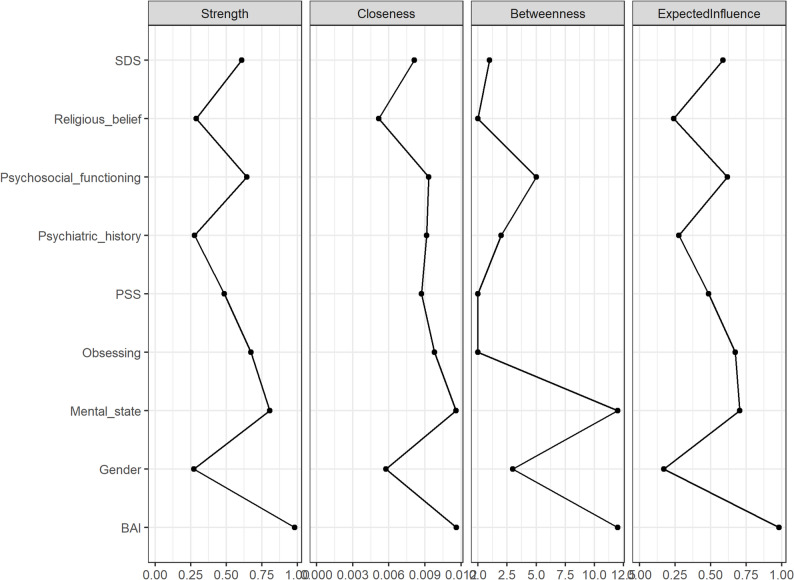
Centrality plot. The centrality estimates show the associations between depression symptom influencing factors within the network. The centrality scores are standardized (range = -1 to 1, mean = 0). Higher scores indicate greater centrality within the network, highlighting the most influential factors in the depression-related network

The centrality plot (Fig. 5.) demonstrated good network stability, with a correlation stability coefficient (CS-C) of 0.750 for node strength, exceeding the recommended threshold for excellent stability (0.50). The strongest connections were observed between BAI and mental state, as well as between psychosocial functioning and SDS. Strength centrality analysis further indicated that BAI occupied the most central position in the network, followed by mental state and obsessing (network-related figures are provided in Supplementary Fig S6–S10).

Finally, we integrated the performance of key variables across three analytical dimensions—predictive importance (SHAP values), statistical associations (odds ratios), and network structure (centrality indices) (Supplementary Table S2, multi-method results alignment table). BAI, psychosocial functioning, mental state, obsessing, and SDS were consistently identified as core features across all approaches. Notably, BAI ranked highest across all three dimensions, highlighting its prominent position within the feature structure characterizing OCD with depressive symptoms.

## Discussion

This nationwide, multi-center study, conducted as part of the Chinese OCD Cohort Research, involved 1,293 outpatients and inpatients across 15 OCD clinic centers in specialized psychiatric hospitals and general hospitals throughout China. By leveraging both the XGBoost model and network analysis, the core factors associated with depressive symptoms in individuals with OCD were systematically identified, and the relationships between these factors were examined. Results indicated that over half of OCD patients (55.7%) exhibited depressive symptoms, with severe depression accounting for 17.2% of cases. This aligns with findings from large-scale epidemiological surveys in the Netherlands, Turkey, and the United States [12, 14–16], underscoring the high prevalence and clinical severity of comorbid OCD and depressive symptoms. Notably, the severity of OCD symptoms appears to be closely related to the risk of developing depression. In addition, logistic regression analysis confirmed the statistical association between individual factors and depressive symptoms, while the XGBoost model further refined the identification of 7 core predictors, which were ranked according to their SHAP values: anxiety (BAI), psychosocial functioning, mental state, functional impairment (SDS), obsessing, perceived stress (PSS), and gender. Higher levels of anxiety, poorer psychosocial functioning, and a more negative self-reported mental state consistently contributed to increased predicted risk of depressive symptoms, highlighting their dominant influence within the model. Network analysis provided complementary insights into how these key features clustered and co-occurred within the OCD population. Several strong associations were observed, particularly between anxiety and obsessing, anxiety and perceived stress, psychosocial functioning and functional impairment, and mental state with both psychosocial functioning and obsessing. Centrality analyses further showed that anxiety occupied the most central position in the network, followed by mental state and obsessing, indicating that these features were most extensively connected to other nodes. The integration of XGBoost and network analysis methods in large-scale epidemiological data offers a robust framework for enhancing the identification of key risk factors associated with depression in OCD, potentially informing future interventions and treatment strategies for OCD patients with depressive symptoms.

Among all predictive variables, anxiety demonstrated the strongest predictive value. SHAP-based interpretation indicated that anxiety contributed most prominently to the model, closely aligning with the high odds ratio observed in the logistic regression analysis (OR = 9.37). Together, these findings suggest that severe anxiety represents the most sensitive indicator for identifying depressive symptoms secondary to OCD. The relationship between anxiety and depression has been extensively examined in psychiatry, with evidence showing that core anxiety features are strongly associated with subsequent risk of developing depression [53–56], and that specific anxiety symptoms exhibit bidirectional and cross-predictive associations with major depressive episodes [57]. In OCD populations, anxiety and depressive symptoms frequently co-occur and are closely intertwined at the symptom level. Elevated anxiety has been linked to greater symptom severity and poorer clinical outcomes among OCD patients with depressive symptoms [17, 24, 58]. Network-based studies further suggest that symptoms such as anhedonia and excessive worry may function as bridge features connecting anxiety and depressive symptom domains [59]. From a neurobiological perspective, anxiety and depression share substantial overlap in both bottom–up emotional processing biases and top–down regulatory dysfunction. Specifically, both conditions are characterized by heightened amygdala reactivity, reflecting increased sensitivity to threat-related or uncertain stimuli [60]. This bottom–up hyperactivation is accompanied by impaired top–down control from the prefrontal cortex, particularly the dorsolateral prefrontal regions, resulting in deficient cognitive regulation of emotional responses [61].

In addition, psychosocial functioning and a negative self-rated mental state were identified as important correlates of depressive symptoms. Psychosocial functioning is closely linked to quality of life and is influenced by multiple clinical and contextual factors [62]. In the present model, poorer overall functioning among individuals with OCD was strongly associated with more severe depressive symptoms, consistent with previous findings [63–66]. Longitudinal evidence further indicates that, compared with the general population and patients with other psychiatric or psychosomatic conditions, individuals with OCD exhibit markedly reduced psychosocial functioning and quality of life, with depressive symptoms substantially exacerbating this functional impairment [67]. Notably, a poorer self-rated mental state showed independent predictive value in the present analysis. This finding suggests that individuals’ global subjective evaluation of their mental state may integrate information across multiple emotional experiences and functional perceptions, thereby serving as a salient marker for identifying elevated risk of depressive symptoms. Previous studies have demonstrated that self-rated mental health is not only strongly associated with concurrent depressive severity but also predicts subsequent emotional deterioration [68]. A lower self-rated mental state may further reflect enduring negative cognitive schemas, characterized by persistent monitoring of internal states and a tendency toward negative interpretation of emotional experiences. Over time, such patterns may contribute to psychological exhaustion and diminished emotion regulation capacity, thereby increasing vulnerability to depressive symptoms [69–72].

Following the identification of key variables associated with depressive status by the XGBoost model, network analysis further characterized their co-occurrence structure within the OCD population. At the global level, anxiety, mental state, obsessing, and psychosocial functioning showed relatively high centrality, indicating close connections with multiple other features. Among these, anxiety emerged as the most prominent node, exhibiting elevated bridge strength and bridge expected impact, which highlights its linking role across different feature domains. Specifically, anxiety showed strong positive associations with obsessing and perceived stress, and a direct connection with self-rated mental state, forming the most salient cluster in the network. This pattern suggests that, in OCD, anxiety is closely embedded within intrusive cognitive experiences and heightened stress sensitivity, rather than functioning as an isolated emotional dimension.

Beyond the anxiety-related configuration, network analysis also identified a highly interconnected substructure centered on psychosocial functioning, functional impairment (SDS), and self-reported mental state. These three features were all positively associated with one another, indicating that, in OCD, impairments in everyday functioning, broader psychosocial maladaptation, and negative self-evaluation of mental state tend to co-vary. Consistent with this pattern, a network study by Heller et al. demonstrated that, in OCD with depressive symptoms, a core symptom cluster comprising compulsive rituals, depressed mood, and anhedonia was strongly linked to poorer psychosocial functioning, including reduced social engagement and interpersonal adaptation, with stronger symptom connectivity corresponding to greater functional impairment [73]. Our previous studies similarly showed that OCD patients with more severe depressive symptoms exhibit more pronounced deficits across multiple domains of psychosocial functioning [27, 74, 75]. Moreover, Zhou et al. reported that individuals with anxiety-dominant depression experience substantial reductions in quality of life alongside lower perceived social support [64]. Taken together, the close alignment between subjective mental state and objective functional indices underscores the structural relevance of functional and experiential dimensions within depression-related phenotypes in OCD.

Although obsessing contributed less directly to the XGBoost predictive model, it exhibited relatively high bridge centrality within the network, indicating a key role in linking distinct psychological domains. Previous network studies have similarly shown that, compared with compulsive behaviors, obsessive thoughts play a more prominent bridging role in connecting OCD with depressive symptoms [76, 77]. Further empirical evidence suggests that specific obsessive dimensions, particularly doubt/checking and symmetry-related obsessions, are significantly associated with higher levels of both depressive and anxiety symptoms [78, 79]. As anxiety sensitivity increases, obsessive–compulsive symptom severity tends to intensify in parallel [80], and obsessional themes characterized by heightened responsibility may induce a persistent state of hypervigilance, thereby exacerbating emotional burden [81]. In this context, recurrent intrusive thoughts may amplify anxiety through ruminative or catastrophizing cognitive processes [82], while the short-term relief achieved through compulsive behaviors paradoxically reinforces a self-perpetuating symptom cycle [83, 84]. In parallel, the network results demonstrated a stable and robust association between anxiety and perceived stress. Prior studies have shown that elevated anxiety is often accompanied by a subjective amplification of external stressors, while stress experiences themselves significantly predict both depressive and anxiety symptoms and are associated with reduced subjective well-being [85–87]. The present network structure further supports the close co-occurrence of anxiety, obsessive cognition, and perceived stress in individuals with OCD. Moreover, higher anxiety levels were significantly associated with more negative self-rated mental state. Existing evidence indicates that sustained anxiety may undermine individuals’ perceived sense of emotional control and intensify negative appraisals of their overall psychological health [88, 89]. Within the current network, anxiety simultaneously connected cognitive symptoms, stress experiences, and subjective evaluations, positioning it as a critical convergence point among multiple depression-related features and underscoring its hub-like role within the network structure.

Both logistic regression and network analysis consistently indicated that male gender was associated with a lower risk of depressive symptoms, which may relate to sex differences in emotion regulation strategies and perceived social support [90–92]. Notably, in our sample, religious affiliation and psychiatric history were both associated with lower odds of depressive symptoms after adjusting for other factors. Evidence from general population studies suggests that religious involvement can be linked to lower prevalence of depressive symptoms and reduced likelihood of previous depression compared with non-religious groups, highlighting the potential protective role of sociocultural support and meaning-making processes (e.g., adjustments for depression and suicide attempts across religious affiliations) [93]. Similarly, qualitative work in obsessive-compulsive disorder has identified religious beliefs as part of broader protective resources against suicidal ideation and emotional distress [94]. In contrast to prior research linking psychiatric history to greater emotional vulnerability and more complex comorbidity patterns [95], our finding suggests a potential protective association in this clinical sample. This discrepancy may reflect differences in clinical engagement, prior treatment exposure, or adaptive coping processes among individuals with previous psychiatric experience, and warrants further investigation. Beyond these background characteristics, developmental factors may further shape emotional vulnerability in OCD. A recent systematic review reported that childhood emotional abuse and neglect were associated with greater OCD severity and a higher prevalence of religious and aggressive obsessions, suggesting that early-life adversity may influence both symptom expression and affective burden in later life [96]. This highlights the importance of considering developmental context when interpreting depressive symptoms and related psychosocial features in OCD.

## Conclusion

In summary, this study adopted an integrated analytical strategy. First, machine learning (XGBoost) was applied to high-dimensional data to identify and rank a core set of features most strongly associated with depressive symptoms in OCD, addressing issues of prediction and feature prioritization. Network analysis was then used to examine the patterns of association among these key features, thereby characterizing their potential synergistic or antagonistic relationships. This combined approach was designed to achieve the dual aims of accurate prediction and a structured understanding of feature interrelationships. Several limitations should be acknowledged. neither the machine learning model nor the network analysis can support causal inference or temporal ordering. All interpretations are now explicitly constrained to contemporaneous relationships observable in cross-sectional data. Second, participants were recruited from specialized clinical centers, which may limit generalizability due to potential selection bias. Third, depressive symptoms were defined based on questionnaire scores rather than standardized clinical diagnoses. Future research should employ longitudinal designs to track symptom trajectories, validate the findings in community-based samples, and integrate multimodal data, such as neurobiological measures, to further evaluate the stability of these associations and explore their underlying mechanisms.

## Supplementary Information

Below is the link to the electronic supplementary material.

Supplementary Material 1

## Data Availability

The data that support the findings of this study are available from the corresponding author upon reasonable request.
