# Supplementary material for: Factors linked to depressive symptoms in obsessive-compulsive disorder: a machine learning and network analysis from China OCD Cohort (COCC)
Source: BMC Psychiatry. 2026 Jan 31;26:184. doi: 10.1186/s12888-026-07854-z (PMC12922223; doi:10.1186/s12888-026-07854-z)
Supplement: Supplementary file 1 — Supplementary Material 1 [file 12888_2026_7854_MOESM1_ESM.docx]

**Factors linked to depressive Symptoms in Obsessive-Compulsive Disorder: A Machine Learning and Network Analysis From China OCD Cohort（COCC）**

**CONTENTS:**

**Methods S1**: Specific workflow of machine learning

**Methods S2**: Network analysis of depression-related factors

**Methods S3**: Protocol for Multi-Method Result Integration Analysis

**Table S1**: Comparison of the related factors of depressive symptoms in patients with obsessive-compulsive disorder and Logistic regression analysis

**Fig. S1**: Boxplot of AUC stability evaluation based on 10 random training-test splits of the model

**Fig. S2**: SHAP dependency plot of BAI

**Fig. S3**: SHAP dependency plot of psychosocial functioning

**Fig. S4**: SHAP dependency plot of mental state

**Fig. S5**: Scatter plot and correlation test results of feature importance ranking consistency between the classification model and regression model (continuous BDI-II outcome)

**Fig. S6**: Stability of centrality parameters

**Fig. S7**: Edge Accuracy

**Fig. S8**:Significant Difference Testing for Edge Weight Estimates

**Fig. S9**: Edge Stability

**Fig. S10**: Comparison of node centrality metrics between the raw network without residualization of confounding variables and the main analysis network

**Table S2**: Multi-method results alignment table

**Supplemental References**

**Methods S1: Specific workflow of machine learning**

**S1.1. Machine learning workflow for identifying factors associated with depressive symptoms**

**S1.1.1. Data preprocessing and definition of study variables**

The primary outcome of this study was defined as the presence of clinically significant depressive symptoms, operationalized as a total score ≥ 14 on the Beck Depression Inventory–II (BDI-II) [1] . This cutoff corresponds to the threshold for at least mild depressive symptoms as defined in the Diagnostic and Statistical Manual of Mental Disorders, Fifth Edition (DSM-5), and has been validated in Chinese populations with good psychometric properties and clear clinical relevance.

All other collected variables, including sociodemographic characteristics, clinical scale scores, and functional measures, were treated as potential predictors (features). Preliminary data inspection indicated that the proportion of missing values for all variables was low (all < 2%). Given the minimal level of missingness and to avoid the additional complexity that multiple imputation might introduce into subsequent machine learning procedures, a conservative single-imputation strategy was adopted: categorical variables were imputed using the mode, and continuous variables were imputed using the mean [2].

To strictly prevent data leakage, the full dataset was first randomly split into a training set (70%) and an independent test set (30%) using stratified sampling to preserve the proportion of depressive cases in both subsets. All imputation parameters (means and modes) were derived exclusively from the training set and then applied unchanged to the test set.

**S1.1.2. Handling class imbalance and feature scaling**

In the present sample, individuals with depressive symptoms accounted for 55.7% of the cohort, indicating mild class imbalance. To reduce potential bias introduced by this imbalance, the Synthetic Minority Over-sampling Technique (SMOTE) was applied exclusively to the training data [3] . SMOTE balances class distributions by generating synthetic samples of the minority class in feature space. After resampling, all continuous features were standardized using a StandardScaler fitted on the SMOTE-balanced training set (mean = 0, standard deviation = 1). The same scaling parameters were subsequently applied to the test set to ensure consistency.

**S1.1.3. Feature selection**

To enhance model interpretability and generalizability, dimensionality reduction was performed using recursive feature elimination with cross-validation (RFECV) [4] . An XGBoost classifier was selected as the core estimator within the RFECV procedure. RFECV applies a backward stepwise elimination strategy, whereby the least important feature in the current feature set—as determined by the model’s feature_importances_ attribute—is removed at each iteration.

The elimination process was allowed to proceed for a maximum of 20 iterations and incorporated an early stopping criterion to prevent overfitting. Specifically, if the mean area under the receiver operating characteristic curve (AUC) obtained from five-fold cross-validation failed to improve across three consecutive iterations (change < 0.001), the procedure was terminated.

Feature selection performance was evaluated using stratified five-fold cross-validation on the training dataset to preserve class proportions, with AUC serving as the primary optimization metric. The optimal feature subset was defined as the set of predictors that maximized the mean cross-validated AUC across the five folds.

**S1.1.4. Hyperparameter optimization and model training**

Hyperparameter optimization was conducted using the Optuna framework[5] . A comprehensive search space was specified for the XGBoost model as follows. For complexity control, the following parameters were explored: max_depth ranging from 3 to 10; min_child_weight ranging from 1 to 10; and gamma sampled from {1e−3, 1e−2, 0.1, 0.5, 1.0} using a log-uniform distribution.

For the learning process, learning_rate was sampled from {0.01, 0.05, 0.1} using a log-uniform distribution, and n_estimators ranged from 100 to 1000 in increments of 100.

For subsampling and column sampling, subsample was set to values between 0.6 and 1.0, and colsample_bytree, colsample_bylevel, and colsample_bynode were each sampled from {0.5, 0.6, 0.7, 0.8, 0.9, 1.0}.

For regularization, reg_alpha and reg_lambda were sampled from {1e−5, 1e−4, 1e−3, 0.01, 0.1, 1.0} using log-uniform distributions.

The optimization objective in Optuna was to maximize the mean area under the receiver operating characteristic curve (AUC) obtained from five-fold stratified cross-validation on the training dataset using the feature set selected by RFECV. A total of 1000 optimization trials were conducted, with unpromising trials terminated early using the MedianPruner to improve computational efficiency.

During model training, the primary internal validation metric was the mean cross-validated AUC and its corresponding 95% confidence interval. After identifying the optimal hyperparameter configuration, the final XGBoost model was retrained on the entire processed training dataset using these optimized parameters.

**S1.1.5. Model evaluation, interpretation, and sensitivity analyses**

The final model was rigorously evaluated on the independent test dataset. Performance metrics included the area under the receiver operating characteristic curve (AUC), the area under the precision–recall curve (AUPRC), accuracy, sensitivity (recall), specificity, precision, F1 score, and Cohen’s kappa coefficient. In addition, calibration curves were generated using Platt scaling to assess the accuracy of predicted probabilities. Classification performance was also reported at the clinical decision threshold that maximized the Youden index (sensitivity + specificity − 1).

To assess model stability, we conducted an additional 10 independent random train–test splits, each maintaining a 70:30 ratio with stratified sampling. The distribution, mean, and standard deviation of the resulting 10 AUC values were reported.

Model interpretability was achieved using SHapley Additive exPlanations (SHAP) values[6] . We generated global SHAP summary (beeswarm) plots and SHAP dependence plots to visualize the overall impact of individual features and potential interaction effects. In addition, local explanation plots were provided for three representative cases with high, moderate, and low predicted risk. To ensure the robustness of feature importance estimates, the stability of SHAP-based rankings for key features (e.g., anxiety and psychosocial functioning) was examined across the 10 random train–test splits. The direction of feature effects indicated by SHAP values was compared with odds ratios (ORs) derived from logistic regression; any discrepancies (e.g., those arising from feature interactions) were addressed in the Results section.

**Sensitivity analysis**. To evaluate the robustness of the primary findings to the definition of depressive symptoms, we conducted a supplementary analysis using an XGBoost regression model with the continuous BDI-II total score as the outcome variable. This model employed the same preprocessing, feature selection (RFECV), and hyperparameter optimization (Optuna) procedures as the classification model. Consistency between the classification and regression models was assessed by comparing the sets and rankings of important features identified by each approach.

**Methods S2: Network analysis of depression-related factors**

**S2.1. Network objectives and node selection**

Network analysis was conducted to characterize the associative structure among key predictive factors jointly identified by the machine learning model and logistic regression. The depressive outcome variable itself was not included as a network node. Instead, the network was designed to depict patterns of co-occurrence and interaction among these predictors within the OCD population.

**S2.2. Network estimation, regularization, and control of confounders**

Networks were estimated using the graphical least absolute shrinkage and selection operator (graphical LASSO, glasso) combined with the extended Bayesian information criterion (EBIC) to enhance sparsity. The EBIC hyperparameter γ was set to 0.25[7, 8] , balancing model fit and complexity penalization to identify stable and interpretable network structures. The network included a mixture of continuous and categorical node variables. To control for potential confounding effects, all continuous variables entered into the network were residualized for age, sex, and OCD symptom severity (total Y-BOCS score) prior to network estimation. As a sensitivity analysis, an additional network was constructed without residualization for confounders, and the centrality indices of core nodes were compared between the residualized and non-residualized networks. This procedure was used to evaluate the impact of confounder control on network structure and to assess the robustness of the main findings. Results indicated that the core topological features of the network (e.g., the three nodes with the highest centrality) remained stable across both approaches, supporting the reliability of the conclusions.

**S2.3. Assessment of network stability and reliability**

Network stability was evaluated using nonparametric bootstrap resampling (1,000 iterations) implemented in the bootnet package[9] . The following aspects were assessed: 1) Bootstrap 95% confidence intervals (CIs) for edge weights. 2) The correlation stability coefficient (CS-C), which quantifies the maximum proportion of cases that can be randomly removed while maintaining a high correlation (r > 0.7) between the centrality indices of the subset network and those of the original network. According to established guidelines, a CS-C ≥ 0.50 indicates excellent stability, whereas a CS-C ≥ 0.25 is considered acceptable. If CS-C < 0.25, the corresponding centrality estimates are deemed unreliable; such cases are explicitly noted in the Results section and not overinterpreted. 3) Network robustness to varying sample sizes, assessed via case-dropping bootstrap procedures.

Centrality metrics computed in the network analysis (e.g., strength, closeness) reflect the degree of connectivity or potential intermediary role of nodes within the network of predictive factors. Given the cross-sectional nature of the data, these indices cannot and should not be interpreted as indicating causal importance for depressive outcomes or as priorities for clinical intervention.

**Methods S3**: Protocol for Multi-Method Result Integration Analysis

To systematically integrate, compare, and interpret the results of the three core analytical methods employed in this study (machine learning predictive modeling, traditional statistical validation, and network relationship exploration), thereby constructing a coherent evidence chain, we have developed a dedicated multi-method result integration analysis protocol. The core of this protocol is the construction of a "multi-method result integration alignment table," which aims to structurally summarize the findings of the progressive analysis rather than directly comparing methodological indicators of distinct natures.

**This alignment table will present the following three categories of core results in parallel:**

**Machine learning predictive importance**: Lists the top 10 most important features selected by the XGBoost model along with their mean absolute SHAP values, so as to quantify the relative contribution of each variable in predicting depressive status.

**Traditional statistical association strength**: Displays the odds ratios (OR) and their 95% confidence intervals (95% CIs) of the aforementioned features in the fully adjusted multivariable logistic regression model, which is used to evaluate the independent association strength between each variable and the depressive outcome after controlling for other confounding factors.

**Network structural characteristics**: Presents the node centrality indicators (mainly strength centrality and closeness centrality) and their correlation stability coefficients (CS-C) of these variables in the "depression-related factor network," thereby describing the connection tightness and mediating potential of each factor within the identified association network of predictive factors.

**The construction of this integration alignment table is mainly based on the following purposes:**

**Integrate the evidence chain**: Clearly and structurally present the complete research evidence from risk factor identification (machine learning/logistic regression) to inter-factor relationship exploration (network analysis), facilitating readers' comprehensive understanding.

**Examine result robustness**: Achieve multi-angle cross-validation of key findings by observing the performance of the same core variable in three different dimensions (predictive importance, independent association strength, and network interaction characteristics). For example, if a variable performs prominently in all three analyses, its importance will receive stronger support.

**Resolve potential inconsistencies**: Provide a systematic comparative framework for identifying and in-depth exploring potential superficial inconsistencies among different methods (e.g., a variable showing a protective association in logistic regression but high centrality in network analysis), thereby guiding reasonable mechanistic explanations in the discussion section.

**Table S1: Comparison of the related factors of depressive symptoms in patients with obsessive-compulsive disorder and Logistic regression analysis.**

| **Independent variable** | **Non-**  **Depression (n =573)** | **Depression (n =720)** | **Odds ratio**  **(95 % CI)** | ***β*** |
| --- | --- | --- | --- | --- |
| Age | 29.25±9.67 | 28.30±8.78 | 1.13(0.95～1.34) | 0.121 |
| Gender (Ref: female) |  |  |  |  |
| Male | 356(62.1%) | 375(52.1%) | 0.43(0.30～0.62) | -0.837^***^ |
| Years of Education | 14.66±2.83 | 14.47±2.85 | 0.99(0.93～1.05) | -0.013 |
| Childhood Rural Residency History (Ref: None) |  |  |  |  |
| Yes | 310(54.1%) | 422(58.6%) | 1.14(0.81～1.60) | 0.132 |
| Primary Caregiver During Childhood (Ref: other) |  |  |  |  |
| Parents | 520(90.8%) | 649(90.1%) | 1.85(0.29～11.87) | 0.617 |
| Single Father | 6(1.0%) | 9(1.3%) | 2.02(0.18～22.58) | 0.701 |
| Single Mother | 18(3.1%) | 26(3.6%) | 2.39(0.31～18.26) | 0.869 |
| Grandparents | 21(3.7%) | 28(3.9%) | 2.40(0.31～18.41) | 0.874 |
| Foster Parents | 3(0.5%) | 1(0.1%) | 0.65(0.01～44.03) | -0.432 |
| Parental Relationship During Childhood  (Ref: Frequent Arguments) |  |  |  |  |
| Very Harmonious | 130(22.7%) | 118(16.4%) | 1.02(0.47～2.22) | 0.017 |
| Harmonious | 269(46.9%) | 301(41.8%) | 0.75(0.38～1.48) | -0.283 |
| Neutral | 69(12.0%) | 120(16.7%) | 0.87(0.42～1.79) | -0.146 |
| Occasional Arguments | 71(12.4%) | 99(13.8%) | 0.658(0.32～1.37) | -0.418 |
| Occupation (Ref: Retired) |  |  |  |  |
| Farmer | 2(0.3%) | 6(0.8%) | 1.88(0.09～37.63) | 0.632 |
| Staff | 258(45.0%) | 300(41.7%) | 1.82(0.22～15.18) | 0.599 |
| Student | 205(35.8%) | 251(34.9%) | 1.24(0.14～11.07) | 0.213 |
| Self-employed | 42(7.3%) | 40(5.6%) | 1.66(0.19～14.94) | 0.507 |
| Unemployed | 61(10.6%) | 120(16.7%) | 1.28(0.15～10.82) | 0.249 |
| Employment Status  (Ref: Unemployed) |  |  |  |  |
| full-time | 424(74.0%) | 462(64.2%) | 0.94(0.62～1.44) | -0.06 |
| Part-time | 22(3.8%) | 23(3.2%) | 0.38(0.16～0.95) | -0.959^*^ |
| Marital Status  (Ref: Never Married) |  |  |  |  |
| Married | 183(31.9%) | 230(31.9%) | 1.19(0.73～1.94) | 0.173 |
| Religious Belief  (Ref: None) |  |  |  |  |
| Yes | 27(4.7%) | 45(6.3%) | 0.44(0.21～0.90) | -0.831^*^ |
| Current Family Situation  (Ref: Frequent Arguments) |  |  |  |  |
| Very Harmonious | 147(25.7%) | 124(17.2%) | 1.02(0.37～2.79) | 0.017 |
| Harmonious | 283(49.4%) | 322(44.7%) | 1.15(0.46～2.88) | 0.138 |
| Neutral | 70(12.2%) | 135(18.8%) | 1.57(0.61～4.04) | 0.449 |
| Occasional Arguments | 58(10.1%) | 97(13.5%) | 1.13(0.43～2.96) | 0.121 |
| Major Life Events in the Last Month (Ref: None) |  |  |  |  |
| Yes | 49(8.6%) | 121(16.8%) | 0.84(0.52～1.37) | -0.172 |
| Mental State (Ref: Good) |  |  |  |  |
| Poor | 84(14.7%) | 374(51.9%) | 3.56(1.86～6.82) | 1.27^***^ |
| Neutral | 366(63.9%) | 320(44.4%) | 1.55(0.86～2.81) | 0.44 |
| PSS | 19.43±4.36 | 22.42±3.89 | 1.05(1.00～1.10) | 0.046^*^ |
| Obsessive-Compulsive Personality Disorder  (Ref: None) |  |  |  |  |
| Yes | 451(78.7%) | 626(86.9%) | 0.99(0.63～1.56) | -0.006 |
| Age of Onset | 22.38±9.29 | 21.23±8.57 | 0.88(0.74～1.05) | -0.128 |
| Course of Illness | 6.87±6.32 | 7.05±6.55 | 0.88(0.74～1.05) | -0.124 |
| Current Illness Form  (Ref: Chronic for Over 3 Months) |  |  |  |  |
| Acute (One Month) | 49(8.6%) | 66(9.2%) | 0.83(0.47～1.46) | -0.188 |
| Subacute | 33(5.8%) | 52(7.2%) | 1.01(0.54～1.87) | 0.006 |
| Illness Course Characteristics  (Ref: Intermittent) |  |  |  |  |
| Continuous | 444(77.5%) | 589(81.8%) | 0.86(0.67～1.31) | -0.147 |
| Delayed Medical Consultation | 4.33±5.48 | 4.18±4.74 | 0.98(0.94～1.02)) | -0.019 |
| Family History of Mental Illness (Ref: None) |  |  |  |  |
| Yes | 83(14.5%) | 98(13.6%) | 1.42(0.89～2.26) | 0.348 |
| Psychiatric History  (Ref: None) |  |  |  |  |
| Yes | 216(37.7%) | 333(53.2%) | 0.65(0.47～0.89) | -0.435^**^ |
| Past Physical Illness History (Ref: None) |  |  |  |  |
| Yes | 64(11.2%) | 102(14.2%) | 0.79(0.43～1.46) | -0.23 |
| Current Physical Illness (Ref: None) |  |  |  |  |
| Yes | 51(8.9%) | 99(13.8%) | 1.32(0.69～2.55) | 0.278 |
| Currently Taking Medication (Ref: None) |  |  |  |  |
| Yes | 419(73.1%) | 493(68.5%) | 1.15(0.79～1.67) | 0.139 |
| Past Medication Use  (Ref: None) |  |  |  |  |
| Yes | 275(48.0%) | 381(52.9%) | 1.16(0.82～1.63) | 0.147 |
| Psychotherapy (Ref: None) |  |  |  |  |
| Yes | 254(44.3%) | 330(45.8%) | 0.92(0.66～1.30) | -0.079 |
| Hospitalization (Ref: None) |  |  |  |  |
| Yes | 116(20.2%) | 136(18.9%) | 1.40(0.90～2.20) | 0.338 |
| OCIR |  |  |  |  |
| Cleaning | 3.74±3.51 | 5.12±3.94 | 1.00(0.95～1.05) | -0.004 |
| Obsessing | 4.28±2.79 | 7.00±2.89 | 1.08(1.01～1.16)) | 0.078^*^ |
| Hoarding | 1.81±2.17 | 2.81±2.58 | 1.04(0.96～1.12) | 0.035 |
| Ordering | 2.54±2.47 | 3.95±3.03 | 1.05(0.97～1.13) | 0.046 |
| Checking | 3.71±3.05 | 5.07±3.28 | 0.98(0.93～1.04) | -0.02 |
| Neutralizing | 2.38±2.62 | 3.70±3.12 | 1.00(0.94～1.07) | 0 |
| Smoking (Ref: No) |  |  |  |  |
| Yes | 87(15.2%) | 116(16.1%) | 0.92(0.56～1.53) | -0.08 |
| Alcohol Consumption  (Ref: No) |  |  |  |  |
| Yes | 163(28.4%) | 220(30.6%) | 0.85(0.58～1.24) | -0.165 |
| YBOCS | 18.39±7.25 | 23.66±6.28 | 1.00(0.97～1.03) | 0.003 |
| Psychosocial Function | 49.75±8.92 | 59.18±8.24 | 1.11(1.08～1.14) | 0.104^***^ |
| SDS | 12.12±6.58 | 18.21±6.01 | 1.07(1.04～1.11) | 0.07^***^ |
| BAI(Ref: None) |  |  |  |  |
| Severe | 21(3.7%) | 192(26.7%) | 9.37(4.93～17.79) | 2.237^***^ |
| Moderate | 87(15.2%) | 244(33.9%) | 4.33(2.74～6.84) | 1.466^***^ |
| Mild | 174(30.4%) | 210(29.2%) | 2.73(1.82～4.11) | 1.004^***^ |

PSS: Perceived Stress Scale; YBOCS: Yale-Brown Obsessive-Compulsive Scale; SDS: Sheehan Disability Scale ; BAI: Beck Anxiety Inventory, The numbers outside and inside the parentheses represent the sample size and percentage (%) for the "non-depressed group" and the "depressed group," respectively. The β represents the regression coefficient. *～p <0.05.**～p <0.01.***～p <0.001

**Fig. S1: Boxplot of AUC stability evaluation based on 10 random training-test splits of the model**


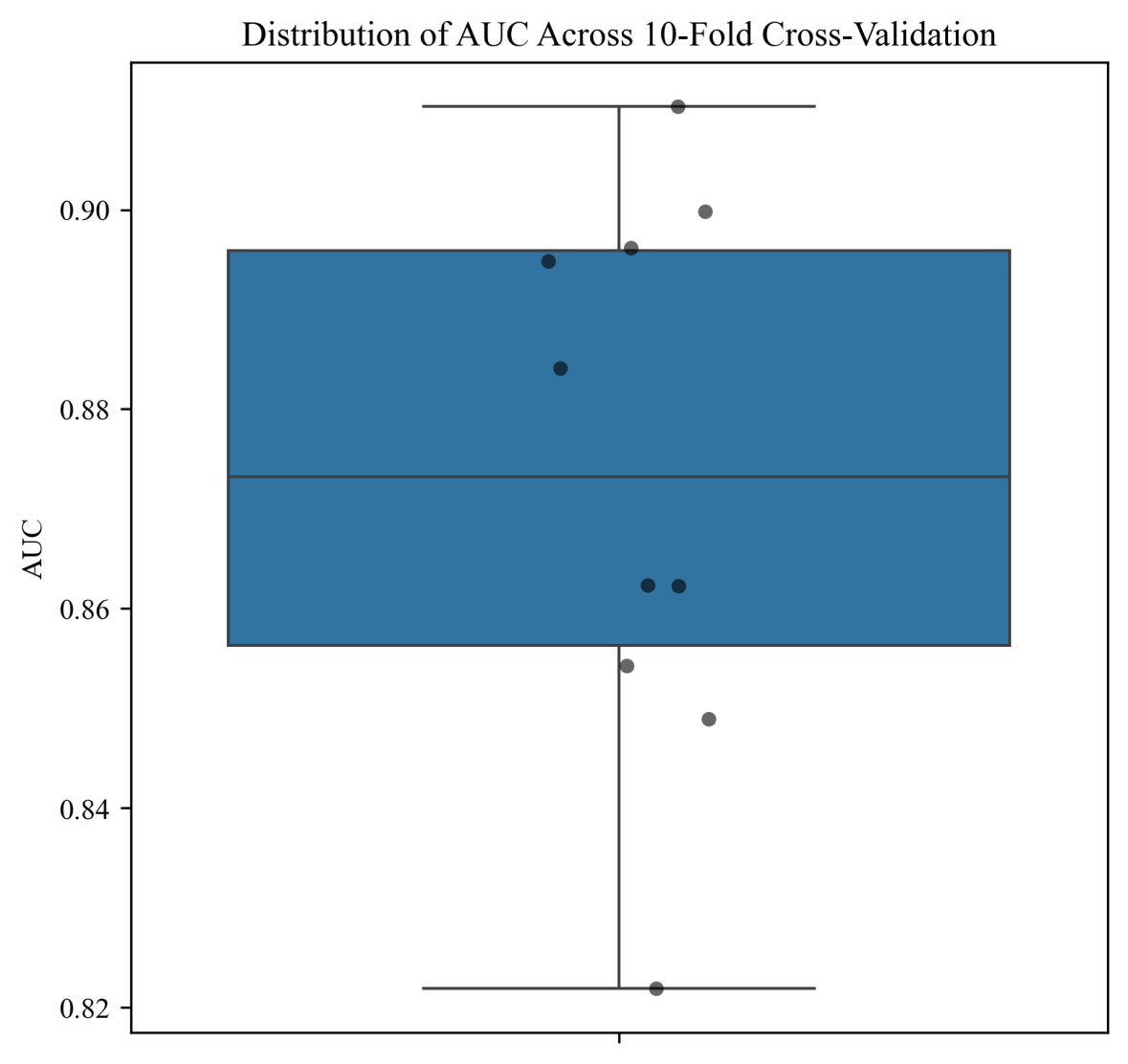


**Fig. S2: SHAP dependency plot of BA**


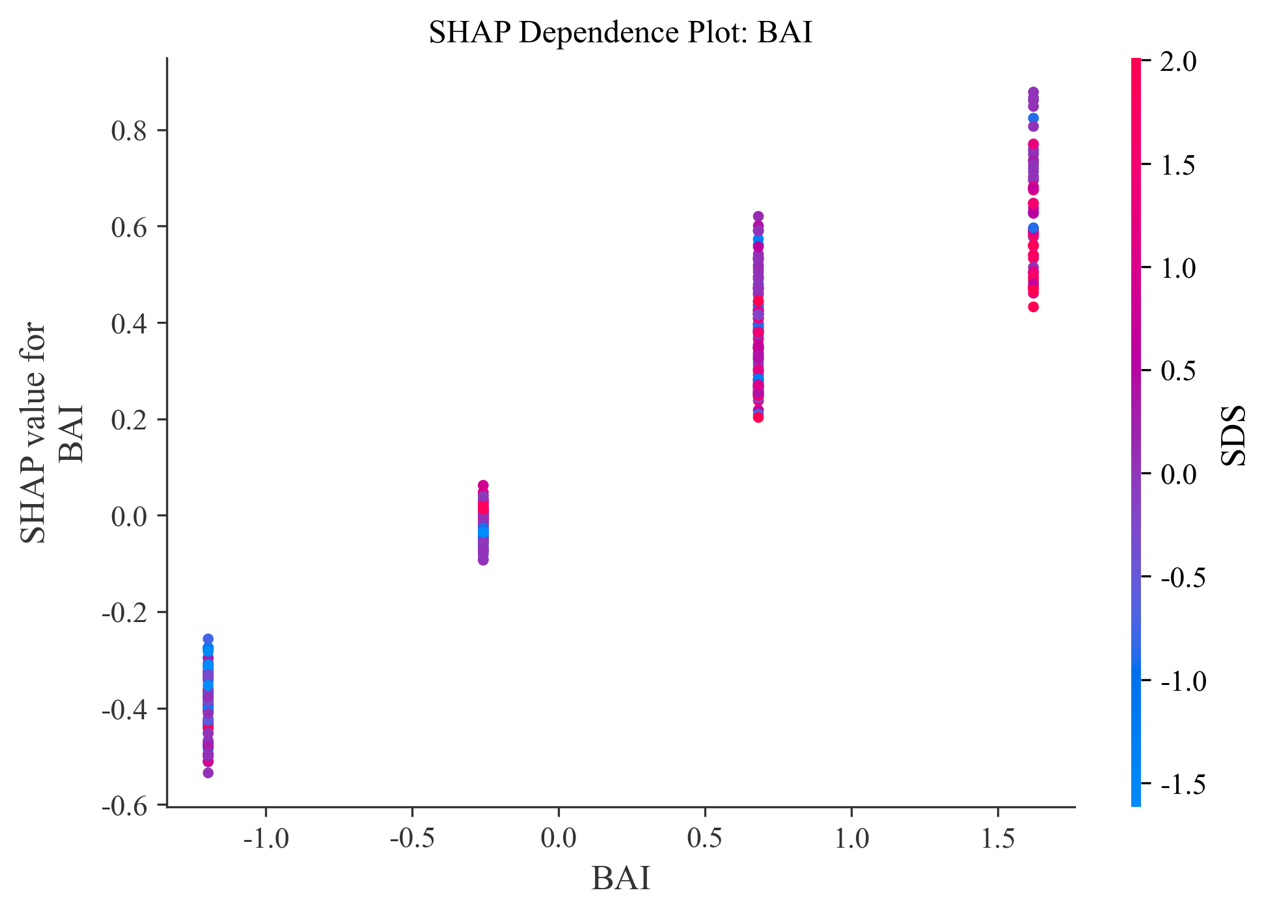


**Fig. S3: SHAP dependency plot of psychosocial functioning**


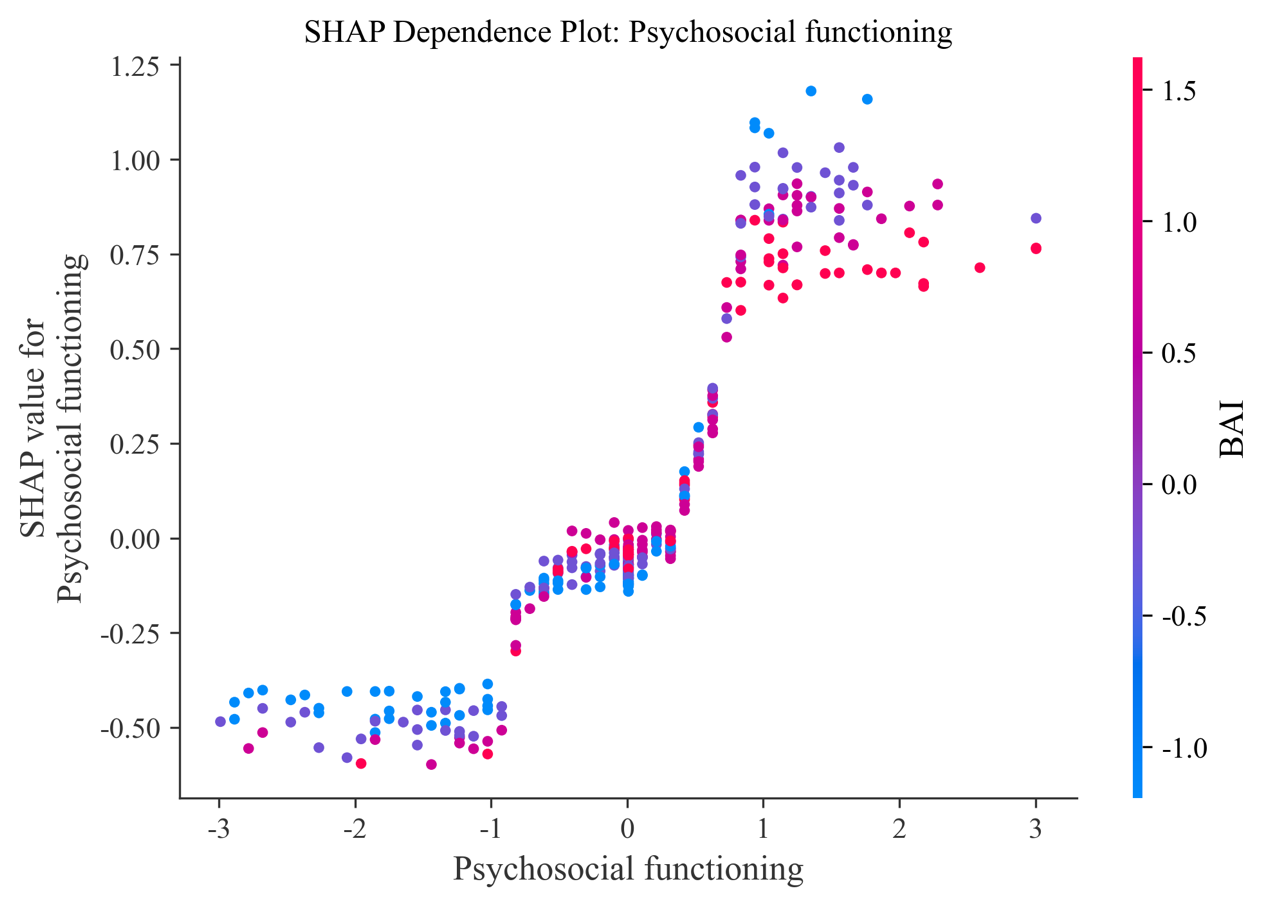


**Fig. S4: SHAP dependency plot of mental state**


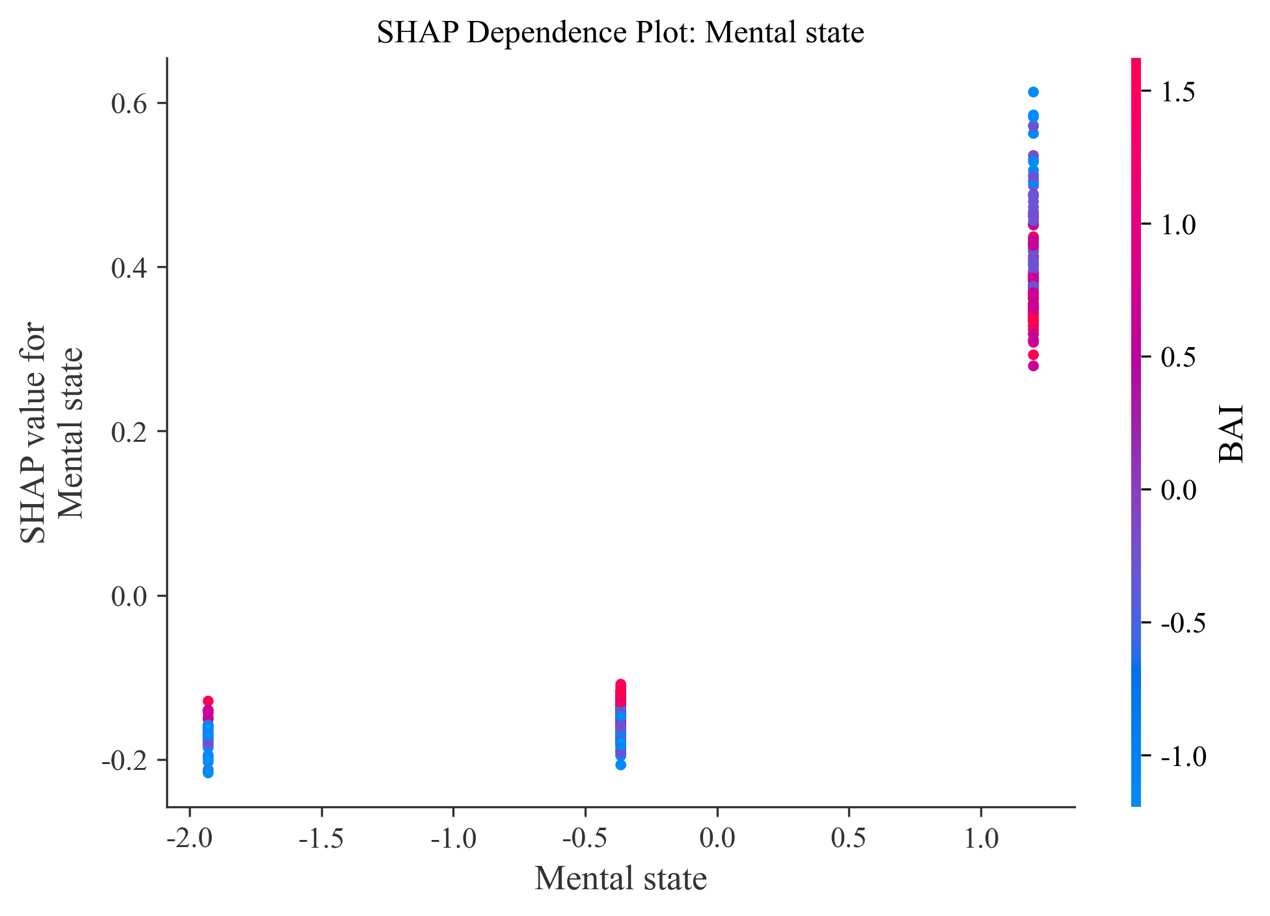


**Fig. S5: Scatter plot and correlation test results of feature importance ranking consistency between the classification model and regression model (continuous BDI-II outcome)**


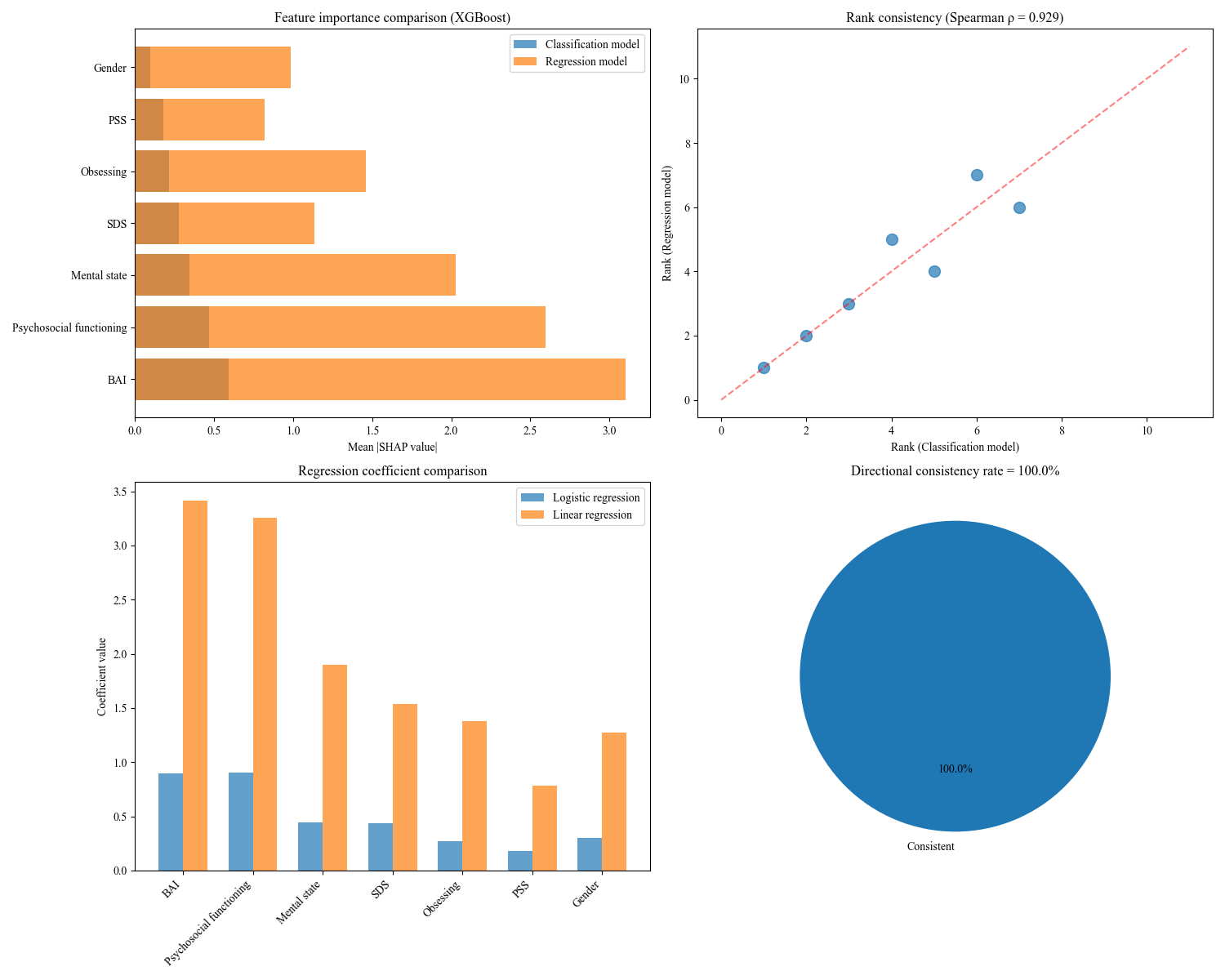


**Fig. S6: Stability of centrality parameters**


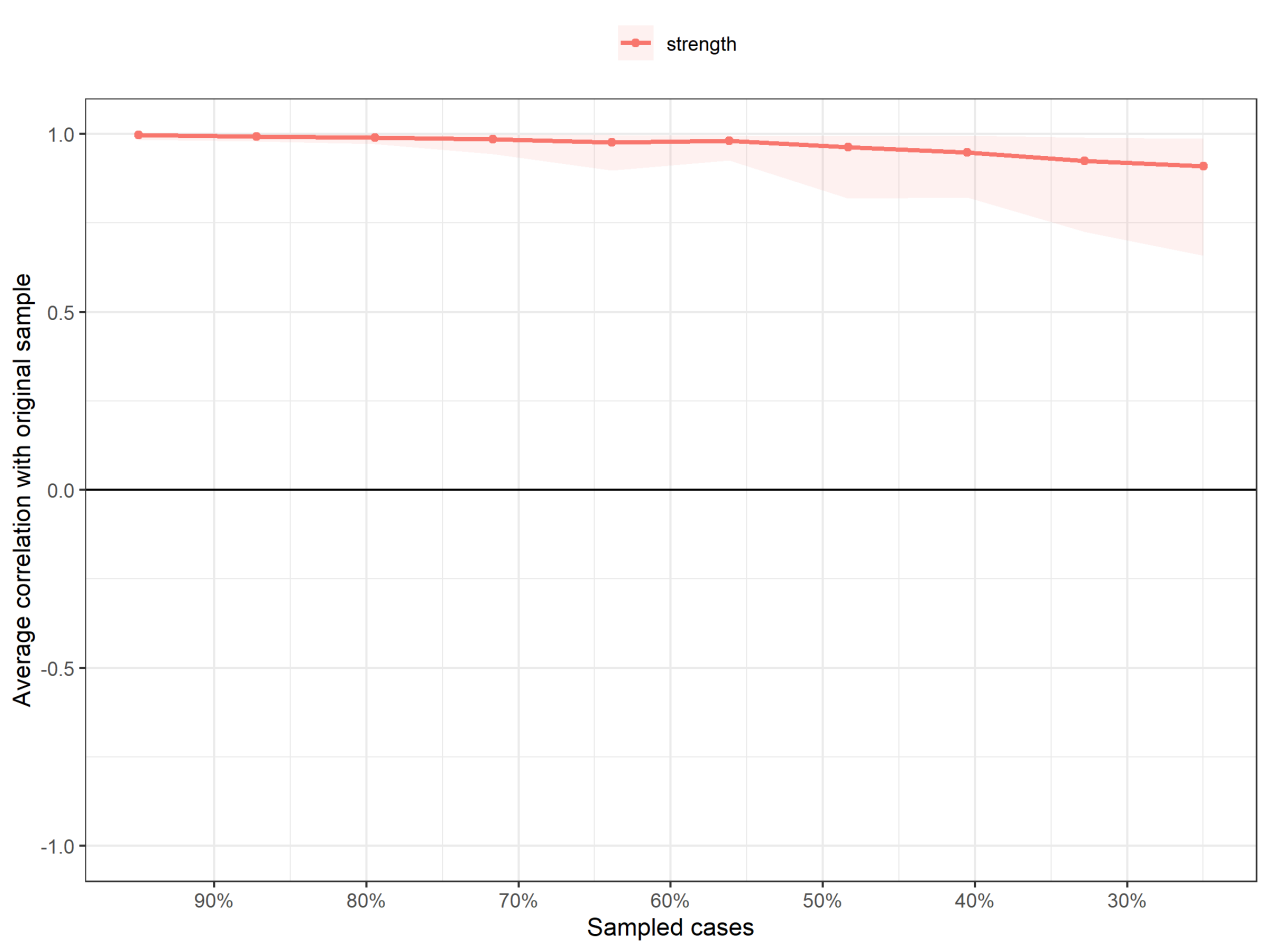


**Fig. S7: Edge Accuracy**


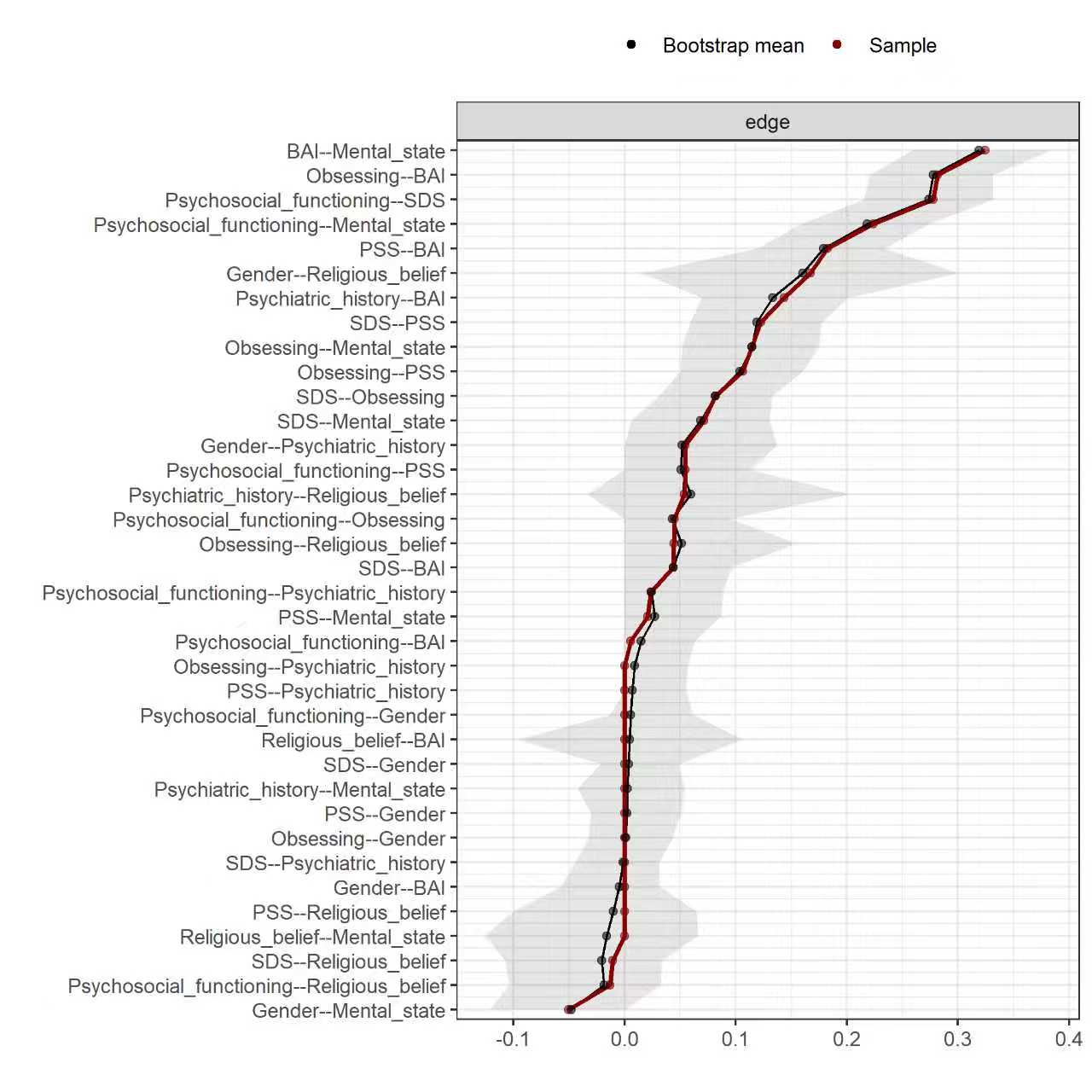


**Fig. S8: Significant Difference Testing for Edge Weight Estimates**


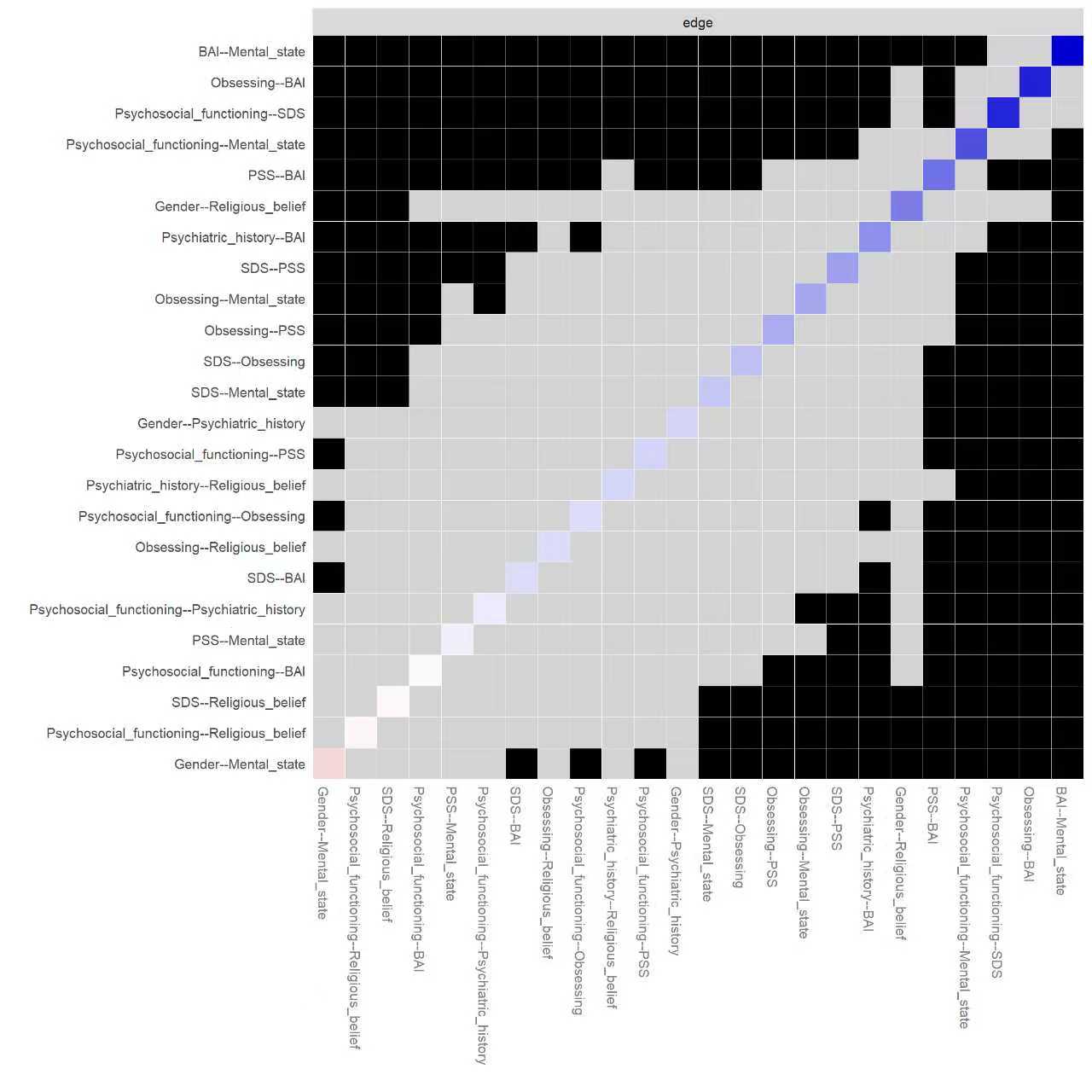


**Fig. S9: Edge Stability**


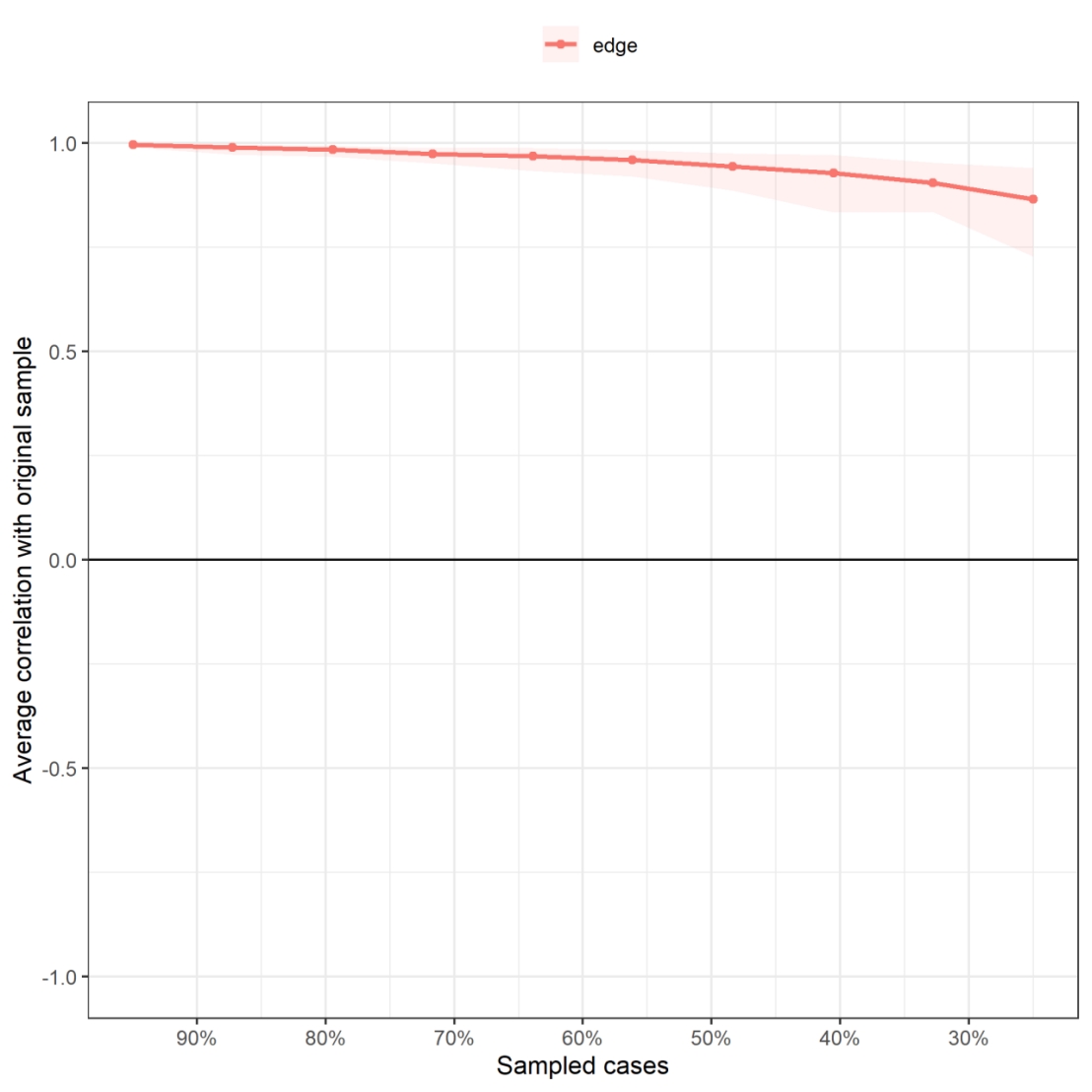


**Fig. S10: Comparison of node centrality metrics between the raw network without residualization of confounding variables and the main analysis network**


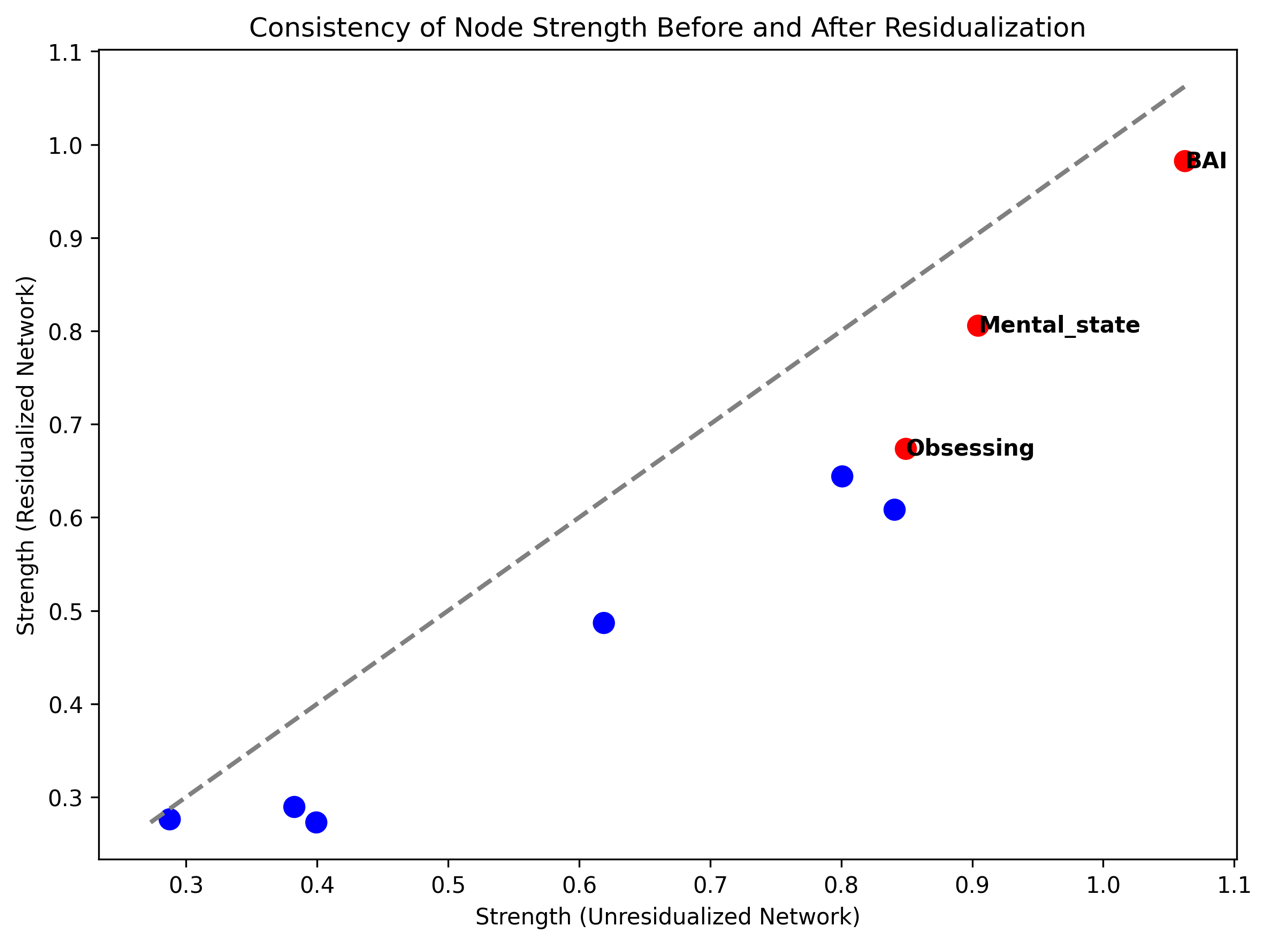


**Table S2: Multi-method results alignment table**

| **variable** | **XGBoost**  **(Average Absolute SHAP Value)** | **logistic regression**  **(OR [95% CI])** | **Network Analysis**  **(Strength Centrality)** | **Evidence integration evaluation** |
| --- | --- | --- | --- | --- |
| **BAI** | 0.316 | 9.37[4.93-17.79] ^***^ | 0.982（max） | Highly Consistent |
| **Psychosocial functioning** | 0.313 | 1.11 [1.08-1.14] ^***^ | 0.644 | Highly Consistent |
| **Mental state** | 0.245 | 3.56 [1.86-6.82] ^***^ | 0.806 | Highly Consistent |
| **Obsessing** | 0.183 | 1.08 [1.01-1.16] ^***^ | 0.674 | Consistent with Prediction and Correlation |
| **SDS** | 0.163 | 1.07 [1.04-1.11] ^*^*^​^* | 0.608 | Consistent with Prediction and Correlation |
| **PSS** | 0.102 | 1.05 [1.00-1.10]^*^ | 0.478 | Consistent with Prediction and Correlation |
| **Gender (male)** | 0.092 | 0.43 [0.30-0.62] ^***^ | 0.273 | Consistent in Direction |

**Supplemental References**

1. Beck AT, Steer RA, Brown GK: BDI-II, Beck depression inventory: manual. *psychological corp* 1996.

2. Munguía T, Armando J: Comparison of Imputation Methods for Handling Missing Categorical Data with Univariate Pattern // Una comparación de métodos de imputación de variables categóricas con patrón univariado. *Revista de Métodos Cuantitativos para la Economía y la Empresa* 2014, 17:101-120.

3. Chawla NV, Bowyer KW, Hall LO, Kegelmeyer WP: SMOTE: synthetic minority over-sampling technique. *J Artif Int Res* 2002, 16(1):321–357.

4. Guyon I, Weston J, Barnhill S, Vapnik V: Gene Selection for Cancer Classification using Support Vector Machines. *Machine Learning* 2002, 46(1):389-422.

5. Akiba T, Sano S, Yanase T, Ohta T, Koyama M: Optuna: A Next-generation Hyperparameter Optimization Framework. *Proceedings of the 25th ACM SIGKDD International Conference on Knowledge Discovery & Data Mining* 2019.

6. Lundberg SM, Lee S-I: A unified approach to interpreting model predictions. In: *Proceedings of the 31st International Conference on Neural Information Processing Systems.* Long Beach, California, USA: Curran Associates Inc.; 2017: 4768–4777.

7. Epskamp S, Fried EI: A tutorial on regularized partial correlation networks. *Psychological methods* 2018, 23(4):617-634.

8. Fried EI, van Borkulo CD, Cramer AO, Boschloo L, Schoevers RA, Borsboom D: Mental disorders as networks of problems: a review of recent insights. *Social psychiatry and psychiatric epidemiology* 2017, 52(1):1-10.

9. Epskamp S, Borsboom D, Fried EI: Estimating psychological networks and their accuracy: A tutorial paper. *Behavior research methods* 2018, 50(1):195-212.
